# The origin of synthons and supramolecular motifs: beyond atoms and functional groups

**DOI:** 10.1107/S2052252525001447

**Published:** 2025-04-07

**Authors:** Rahul Shukla, Emmanuel Aubert, Mariya Brezgunova, Sébastien Lebègue, Marc Fourmigué, Enrique Espinosa

**Affiliations:** a CRM2 Université de Lorraine, CNRS NancyF-54500 France; b Department of Chemistry (NCI Laboratory), GITAM (Deemed to be University), 530045Visakhapatnam, Andhra Pradesh, India; c Institut des Sciences Chimiques de Rennes Université Rennes 1, UMR CNRS 6226 Campus de Beaulieu35042 France; Universidad de Oviedo, Spain

**Keywords:** chalcogen bonding, halogen bonding, hydrogen bonding, electrophilic–nucleophilic interactions, electron density, Laplacian of electron density, topological analysis, crystal engineering

## Abstract

A four-membered *R*_2_^2^(4) supramolecular motif formed by S⋯S and S⋯I chalcogen-bonding interactions in the crystal structure of 4-iodo-1,3-di­thiol-2-one (C_3_HIOS_2_, IDT) is analysed and compared with a similar *R*_2_^2^(4) motif (stabilized by Se⋯Se and Se⋯O chalcogen bonds) observed in the previously reported crystal structure of selena­phthalic anhydride (C_8_H_4_O_2_Se, SePA) through detailed charge density analysis. Our investigation reveals that the chalcogen-bonding interactions participating in the *R*_2_^2^(4) motifs observed in the two structures have the same characteristic orientation of local electrostatic electrophilic⋯nucleophilic interactions while involving different types of atoms. We carried out Cambridge Structural Database searches for synthons and supramolecular motifs involving chalcogen-, halogen- and hydrogen-bonding (ChB, XB and HB) interactions. Geometrical characterizations and topological analyses of the electron density ρ(**r**) and its negative Laplacian function [*L*(**r**) = −∇^2^ρ(**r**)] indicate that all the bonding interactions forming the motifs are driven by local electrophilic⋯nucleophilic interactions between complementary charge concentration (CC) and charge depletion (CD) sites present in the valence shells of the atoms, regardless of the atoms and functional groups involved. The graph-set assignment *G*_*d*_^*a*^(*n*) (*G* = *C*, *R*, *D* or *S*), formerly developed by Etter [*Acc. Chem. Res.* (1990), **23**, 120–126] for HB interactions, is a convenient way to describe the connectivity in supramolecular motifs based on electrophilic⋯nucleophilic interactions (such as ChB, XB and HB interactions), exchanging the number of atomic acceptors (*a*) and donors (*d*) with the number of nucleophilic (*n*: CC) and electrophilic (*e*: CD) sites, and the number of atoms building the motif *n* by *m*, leading to the new graph-set assignment *G*_*e*_^*n*^(*m*) (*G* = *C*, *R*, *D* or *S*). Geometrical preferences in the molecular assembly of synthons and other supramolecular motifs are governed by the relative positions of CC and CD sites through CC⋯CD interactions that, in most cases, align with the internuclear directions within a <15° range despite low interaction energies. Accordingly, beyond atoms and functional groups, the origin of recurrent supramolecular structures embedded within different molecular environments is found in the local electrostatic complementarity of electrophilic and nucleophilic regions that are placed at particular geometries, driving the formation and the geometry of synthons and supramolecular motifs by directional and locally stabilizing electrostatic interactions.

## Abstract 2

This study establishes that hydrogen-, halogen- and chalcogen-bonding intermolecular and non-covalent intramolecular interactions are driven by a face-to-face orientation of electrophilic (charge-depleted) and nucleophilic (charge-concentrated) regions, which is the origin of the specific geometries found in synthons and supramolecular motifs.

## 1. Introduction

Hydrogen bonding is the most commonly observed intermolecular interaction in crystals of organic compounds containing hydrogen atoms of acidic character. The use of rules about the formation of hydrogen bonds can aid in the design and analysis of multi-component crystals of organic compounds, such as co-crystals (Tiekink & Zukerman-Schpector, 2017 ▸). However, so far, the formation of a co-crystal cannot be predicted using these rules, and it can only be verified after experimental analysis. Strategies put in place to build up co-crystals are mainly based on the formation of supramolecular building blocks called synthons (Desiraju, 1995 ▸), which are defined as recurring intermolecular structural units found in crystal phases. Hence, studies on the competition between different supramolecular building blocks have been carried out to facilitate the prediction of co-crystal formation (Etter, 1991 ▸). The results of these studies suggest that the most energetically stable synthon is the one that appears in the arrangement of the co-crystal formed (Blagden *et al.*, 2008 ▸). These hypotheses have been verified experimentally through comparisons of single-crystal structures (Vishweshwar *et al.*, 2006 ▸).

A study carried out using the Cambridge Structural Database (CSD) (Allen, 2002 ▸) and the *CONQUEST* (version 1.17) software demonstrated that most co-crystals form preferentially by making use of heterosynthons rather than homosynthons (Groom & Allen, 2014 ▸). An homosynthon is characterized by intermolecular interactions between the same functional group, such as carb­oxy­lic acid⋯carb­oxy­lic acid or amide⋯amide interactions. An heterosynthon is characterized by intermolecular interactions between different but complementary functional groups, such as carb­oxy­lic acid⋯amide or carb­oxy­lic acid⋯pyridine. Since carb­oxy­lic acids are found in a large number of organic molecules and active pharmaceutical ingredients, they are among the most studied functional groups in crystal engineering (Desiraju, 1991 ▸; Shan *et al.*, 2002*a* ▸; Shan *et al.*, 2002*b* ▸; Vishweshwar *et al.*, 2005 ▸). Statistical analyses carried out using the CSD (Allen *et al.*, 1999 ▸) and involving structures that only contain carb­oxy­lic acid functional groups have shown that the main molecular association follows the formation of the supramolecular homosynthon carb­oxy­lic acid⋯carb­oxy­lic acid. However, in the presence of competing functional groups such as amide or pyridine groups, carb­oxy­lic acid⋯pyridine and carb­oxy­lic acid⋯amide heterosynthons were found to be more favoured (Steiner, 2001 ▸; Vishweshwar *et al.*, 2003*a* ▸; Vishweshwar *et al.*, 2003*b* ▸; Nangia, 2010 ▸). This empirical information on the complementarity between functional groups and their supramolecular building blocks could lead to guidelines for the design of co-crystals. Such guidelines would facilitate the selection of co-formers (namely, the appropriate molecules participating in the formation of co-crystals), which could then be used in experimental screens targeting forms of multi-component solids (Issa, 2011 ▸). The formation of co-crystals can be induced through the use of hydrogen-bond donors and acceptors that are part of the molecular entities forming the co-crystal, as well as the way they may interact through hydrogen bonding (Blagden *et al.*, 2007 ▸). After a thorough study of preferential arrangements and HB patterns found in a large number of organic crystals, Etter made several observations to aid in the design of hydrogen bonds in solids and applied a graph-set assignment to define the morphology of hydrogen-bonded arrays differentiating the type of donors and acceptors that are present (Etter, 1990 ▸). These graph-set symbols are of the form 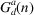, where *G* = *C*, *R*, *D* or *S*, denoting chain, ring, dimer (or other finite set) or intramolecular HB patterns, respectively, while the subscript *d* and superscript *a* indicate the number of donors and acceptors used in each motif, and *n* is the number of atoms in the unit.

The graph-set assignment 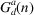 is a very convenient way to describe the connectivity in supramolecular motifs based on electrophilic⋯nucleophilic interactions, generalizing the description of HB patterns to additional ones that originate from other types of non-covalent interactions. Indeed, after the initial use of synthons based on HB interactions, other supramolecular building units have been identified based on halogen bonds (Cavallo *et al.*, 2016 ▸; Fourmigué, 2009 ▸), and more recently on chalcogen bonds (Scilabra *et al.*, 2019 ▸; Vogel *et al.*, 2019 ▸; Gleiter *et al.*, 2018 ▸; Dhaka *et al.*, 2024 ▸; Dukhnovsky *et al.*, 2024 ▸). In all these cases, new supramolecular architectures exhibit specific electrophilic and nucleophilic regions at donor and acceptor molecules that lead to highly directional interactions. Electrophilic (charge-depleted: CD) sites in halogen bonds and chalcogen bonds are associated with σ-hole regions (Clark *et al.*, 2007 ▸; Politzer & Murray, 2017 ▸) and are typically revealed by a plot of the molecular electrostatic potential (MESP) on isosurfaces of electron density in the external parts of molecules, which become significantly positive, as with acidic hydrogen atoms that are involved in HB interactions. On the other hand, good nucleophilic (charge concentration: CC) sites are typically associated with lone-pair regions of Lewis bases involved in HB, XB and ChB interactions, displaying negative MESP values and therefore ensuring the electrostatic complementarity with donor partners. Although additional types of non-covalent electrophilic⋯nucleophilic interactions have been described in the literature (Alkorta *et al.*, 2020 ▸), this work will focus on ChB and XB interactions, and HB interactions to a lesser extent.

The IUPAC defined the chalcogen bond as a net ‘*attractive interaction between an electrophilic region associated with a chalcogen atom in a molecular entity and a nucleophilic region in another, or the same, molecular entity*’ (Aakeroy *et al.*, 2019 ▸). While similar to the halogen bond as per the definition (Desiraju *et al.*, 2013 ▸), characterizing a chalcogen bond can be complex in comparison. This is because the anisotropic distribution of ρ(**r**) around the Ch_*sp*^3^_ atom can result in multiple CD sites suitable for ChB interactions, which can compete and sometimes merge (Shukla *et al.*, 2020 ▸). In comparison, halogen atoms have only one CD region suitable for an XB interaction. This is the reason why intermolecular chalcogen bonds do not always form along the covalent bond direction of the chalcogen atom, as observed in halogen bonds. In the past few years, there has been a steady increase in both experimental and theoretical studies focusing on ChB interactions. This is because, in addition to their ability to form strong intermolecular and intramolecular interactions in crystal structures, chalcogen bonds also present potential applications in the fields of catalysis, organic synthesis and material design, among other fields (Mahmudov *et al.*, 2017 ▸; Bamberger *et al.*, 2019 ▸; Dhaka *et al.*, 2020 ▸; Beau *et al.*, 2023 ▸). The formation of supramolecular synthons mediated by ChB interactions is also a very common phenomenon. However, this feature is relatively less explored compared with motifs involving hydrogen and halogen bonds. Investigations on chalcogenated molecules have shown the formation of robust Ch⋯O interactions with the electrophilicity of the chalcogen atom increasing in the order O < S < Se < Te (Brezgunova *et al.*, 2013 ▸), which parallels the observed behaviour of *X*⋯O interactions along the series of halogen atoms F < Cl < Br < I (Geboes *et al.*, 2015 ▸). Other studies have shown that the characteristics of the Ch⋯O interaction remained similar (Scilabra *et al.*, 2019 ▸) even when one type of chalcogen atom was replaced with another.

In the current study, the crystal structure of 4-iodo-1,3-di­thiol-2-one (C_3_HIOS_2_, hereafter called IDT) is investigated via theoretical charge density analysis. The presence of hydrogen, chalcogen and halogen atoms in the small IDT molecule allows an in-depth investigation of HB, ChB and XB interactions present in the crystal structure of this molecule. A significant structural aspect of IDT on which we focus is the formation of a four-membered motif assembled from S⋯S and S⋯I chalcogen bonds. Considering the expected positions of electrophilic and nucleophilic regions around the atoms involved, the motif in IDT is extremely similar to the recurrent 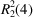 motif in selena­phthalic anhydride (SePA) (Brezgunova *et al.*, 2013 ▸), which is formed by Se⋯Se and Se⋯O chalcogen bonds (see the scheme below[Chem scheme1]). This feature raises the question of the origin of similar supramolecular motifs involving different atoms. Is the origin of the motif associated with the atom or the functional group involved in the formation of the interactions building the supramolecular structure, or with the appropriate orientation of particular electrophilic and nucleophilic regions present in the interacting atoms? The formation of a similar motif in IDT and SePA strongly suggests that the latter holds true. Hereafter, the graph-set assignment 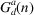 developed by Etter (1990 ▸) for HB interactions is generalized to any non-covalent interaction (such as HB, XB or ChB interactions) using the new notation 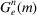 (*G* = *C*, *R*, *D* or *S*), where the number of atomic acceptors *a* and donors *d* are exchanged for the number of nucleophilic *n* (CC) and electrophilic *e* (CD) sites, and the number of atoms building the motif *n* for *m* (to avoid a misunderstanding with the number of nucleophiles).[Chem scheme1]
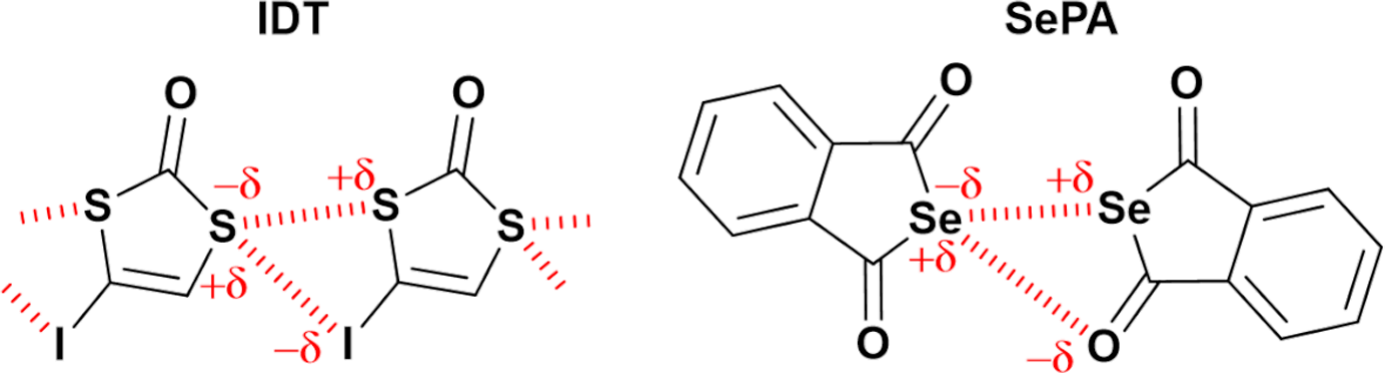


With the aim of identifying the structural parameters that correspond to the geometrical preferences shown in synthons (and in a more general way, in the formation of structurally similar non-covalent interactions), a detailed CSD search has been carried out for other supramolecular 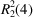, 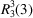 and bifurcated intermolecular motifs, as well as for intramolecular motifs. In addition, to investigate the origin of recurrent non-covalent inter-/intramolecular motifs and their geometries in depth, the topology of *L*(**r**) = −∇^2^ρ(**r**) has been also investigated for selected motifs to determine local nucleophilic (CC) and electrophilic (charge depletion, CD) sites in the valence shells of the atoms and to quantify their geometric orientation with respect to internuclear directions; this procedure has already been applied in previous work and described by the angle between the CC⋯CD and internuclear directions (Brezgunova *et al.*, 2013 ▸; Shukla *et al.*, 2020 ▸). Overall, the investigation carried out in this study establishes that face-to-face CC⋯CD interactions drive atomic orientations and are definitively more important than the atoms or functional groups involved in the formation of different supramolecular synthons or motifs.

## 2. Experimental and theoretical methods

### 2.1. Synthesis and crystal growth

4-Iodo-1,3-di­thiol-2-one (C_3_HIOS_2_, IDT) was synthesized from the corresponding thione 4-iodo-1,3-di­thiol-2-thione (Le Gal *et al.*, 2016 ▸) in CH_3_Cl/AcOH with Hg(OAc)_2_, as shown in earlier work (Domercq *et al.*, 2001 ▸). The sublimation method was used to obtain IDT single crystals of good quality (70°C sublimation temperature).

### 2.2. X-ray diffraction

Low-temperature high-resolution single-crystal X-ray diffraction measurements were performed using an Mo *K*α radiation source (λ = 0.71073 Å) on an Enraf-Nonius diffractometer with a Kappa-CCD detector at *T* = 100 (4) K (Oxford Cryosystems N_2_ cooling device). The data collection strategy was determined and data reduction was carried out with *CrysAlisProCCD*/*RED*. After absorption correction, the measured reflections were then sorted, scaled and merged using the *SORTAV* program (Blessing, 1995 ▸). The structures were solved with *SHELXS* (Sheldrick, 2008 ▸) and refined with *SHELXL* (Sheldrick, 2015 ▸) incorporated in the *OLEX2* program (Dolomanov *et al.*, 2009 ▸). The molecular packing of IDT was drawn using *Mercury* (Macrae *et al.*, 2020 ▸). See Table S1 in the supporting information for data collection and structure refinement details.

### 2.3. Computational electron density

Owing to the presence of several heavy atoms (namely, two sulfur atoms and one iodine atom) in a small organic molecule that crystallizes in the triclinic system with the non-centrosymmetric space group *P*1, the experimental dataset for IDT was of limited quality for experimental charge density analysis (Fig. S1 of the supporting information). For this reason, only periodic theoretical calculations were performed on the crystal structure of IDT based on the experimental geometry and obtained after the independent atom model (IAM) refinement. *Ab initio* periodic calculations using density functional theory (DFT) were then performed on the crystal structure of IDT with the *Vienna Ab initio Simulation Package* (*VASP*) (Kresse & Furthmüller, 1996*a* ▸; Kresse & Hafner, 1993 ▸; Kresse & Hafner, 1994 ▸; Kresse & Furthmüller, 1996*b* ▸). More precisely, we used the functional of Perdew, Burke and Ernzerhof (Perdew *et al.*, 1996 ▸) as the exchange–correlation functional, and to ensure convergence a value of 900 eV was set for the plane-wave expansion of the wavefunction, as well as a *k*-points grid of 16 × 12 × 8 to sample the first Brillouin zone. The valence electron density was represented on a regular grid comprising 160 × 224 × 280 points along the crystallographic axes and was used to compute the structure factors. Note that the comparison of a previous experimental charge density model for C_6_Cl_6_ with the corresponding periodic *ab initio* theoretical DFT model obtained with *VASP*, using the same method as in the present work, led to extremely close results in intermolecular regions (Aubert *et al.*, 2011 ▸).

DFT calculations were also undertaken at the B3LYP/Def2TZVPP level of theory on isolated molecules (monomers, dimer, trimers) in order to characterize the intermolecular interactions. The molecular structures were extracted from crystal structures and the *Gaussian 16* program package was used for the calculations (Frisch *et al.*, 2019 ▸). Topological analyses were also performed using *AIMAll* (Keith, 2019 ▸).

### 2.4. Multipolar refinement

**Figure 1:**
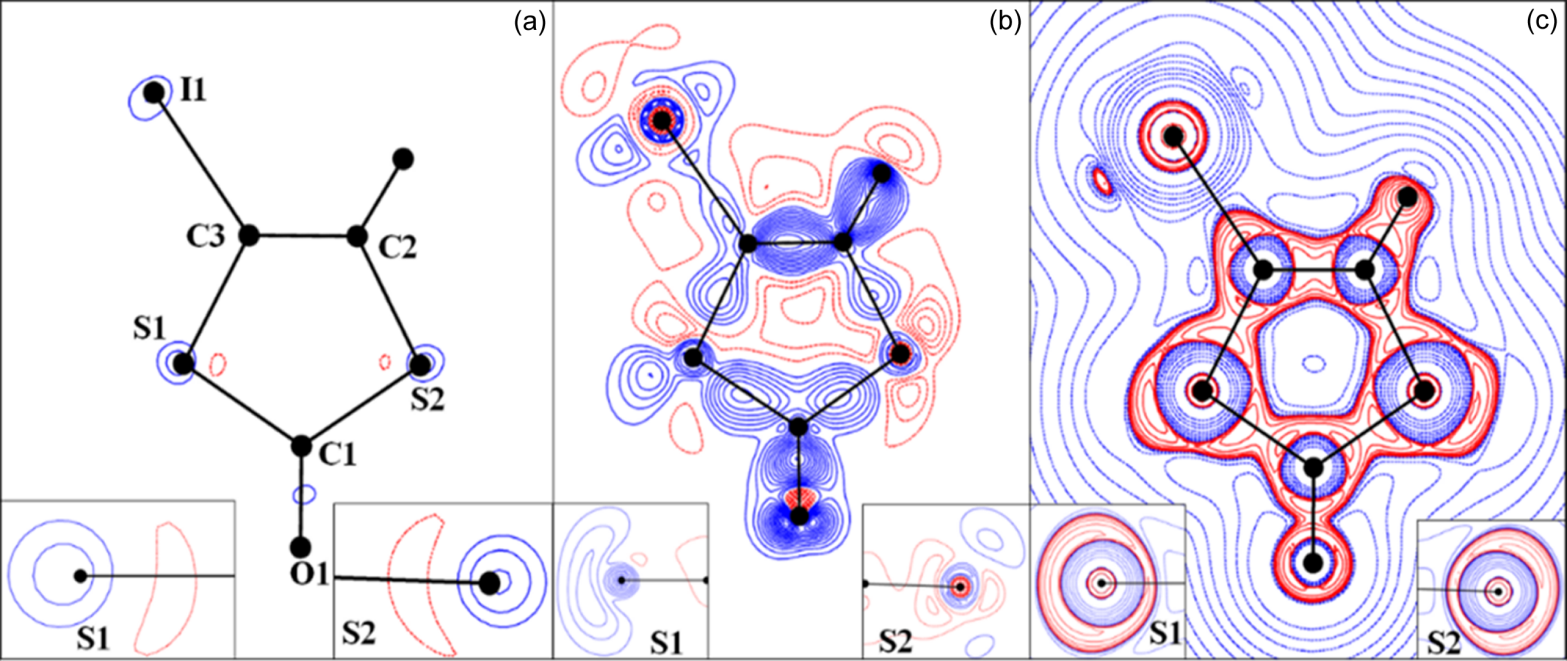
Maps calculated in the molecular plane and in the two perpendicular planes bis­ecting the C—S—C angles containing the S-atom lone pairs (inset figures) of IDT (theoretical model): (*a*) residual electron density at the resolution range 0 ≤ (sin θ/λ) ≤ 0.9 Å^−1^, (*b*) static deformation density Δρ(**r**) and (*c*) *L*(**r**) = −∇^2^ρ(**r**). Contours for maps (*a*) and (*b*) are at the ±0.05 e Å^−3^ level: full lines in blue are positive, dotted lines in red are negative. Contours for (*c*) in e Å^−5^ are on a logarithmic scale: red lines are positive and blue lines are negative.

The theoretical multipolar refinement was performed using the *MoPro*/*MoProviewer* software package (Jelsch *et al.*, 2005 ▸; Guillot *et al.*, 2014 ▸). In the multipolar refinements against the theoretically calculated structure factors (sin θ/λ_max_ = 1.0 Å^−1^), the Slater-type radial functions were expanded up to the hexadecapole level (*l* = 4) for the I (*n_l_*, ξ: 8, 8, 8, 8 for *l* = 1, 2, 3, 4; 4.84 bohr^−1^) and S atoms (*n_l_*, ξ: 4, 4, 6, 8 for *l* = 1, 2, 3, 4; 3.8513 bohr^−1^), and up to octapole level (*l* = 3) for C (*n_l_*, ξ: 2, 2, 3 for *l* = 1, 2, 3; 3.0 bohr^−1^) and O atoms (*n_l_*, ξ: 2, 2, 3 for *l* = 1, 2, 3; 4.5 bohr^−1^). In the fitting against theoretical data, core electrons were omitted from the *MoPro* atomic definitions, since the *VASP* structure factors correspond to valence electrons only. The multipolar models fitted against the theoretical structure factors converged to the agreement factors *wR*(*F*) = 0.023 and *R*(*F*) = 0.041 (Table S1). The residual density map of the best theoretical model shows residual peaks centred at the I and S atoms [Fig. 1 ▸(*a*)]. In the same plane, the static deformation density Δρ(**r**) and *L*(**r**) = −∇^2^ρ(**r**) maps of the theoretical multipolar model of IDT are shown in Figs. 1 ▸(*b*) and 1 ▸(*c*), respectively. The Δρ(**r**) map shows that the highest density accumulation is on the C2—C3 bond (0.6 e Å^−3^) and three is a polarized electron distribution towards the C atoms along the C—S and C—I bonds. The lone pairs of S1 merge in a banana-shaped distribution (their separation is not observed at any contour level), whereas those of S2 are separated [see inset figures in Fig. 1 ▸(*b*)]. However, it is noticeable that a polarization of the electron density takes place towards one of the lone pairs in both S atoms. This effect could be explained by the participation of the S atoms in different intermolecular interactions above and below the molecular plane (S1⋯O1, S2⋯O1 and S1⋯I1 contacts, which result in the stacking of the molecules in columns). The multipolar model shows the depletion of the electron density (δ^+^) extending along the C—S bonds (Δρ varies from −0.25 to −0.10 e Å^−3^). In general, the deformation density map of the S atom exhibits very similar features to those observed earlier for the Se atom in seleno­phtalic anhydride SePA (Brezgunova *et al.*, 2013 ▸). On the other hand, the deformation density of the I atom in IDT is qualitatively close to those previously described for Cl and Br atoms (Bui *et al.*, 2009 ▸; Aubert *et al.*, 2011 ▸; Brezgunova *et al.*, 2012 ▸), namely a δ^+^ region on iodine located on the prolongation of the C3—I1 bond and δ^−^ regions in the plane perpendicular to the bonding direction.

### 2.5. The topology of ρ(**r**) and *L*(**r**) functions

Within the QTAIM framework (Bader, 1990 ▸), topological analysis of ρ(**r**) is performed in order to extract significant information on bonding interactions present in molecular crystals. Accordingly, we have evaluated the topological and local energetic properties of ρ(**r**) at bond critical points (BCPs) for a detailed description of the intermolecular and non-covalent intramolecular interactions investigated in this study. Topological analysis of *L*(**r**) = −∇^2^ρ(**r**), with the determination of its own CPs [different from those of ρ(**r**)], has also been performed in order to identify CD and CC regions present in the valence shells of the atoms participating in the interactions. The local electrostatic complementarity of CD and CC regions interacting through space will help in characterizing the electrophilic–nucleophilic interactions (CD⋯CC) which are the origin of preferred orientations in molecular assemblies.

In the valence shells of atoms, (3,−3)/(3,+3) CPs correspond to 3D nucleophilic/electrophilic sites [the *L*(**r**) distribution is locally concentrated/depleted at the CP along the three main directions], whereas the interpretation of saddle (3,+1)/(3,−1) CPs remains more complex because it depends on the relative positions of other (3,−3)/(3,−1)/(3,+1)/(3,+3) CPs around them. This feature is a consequence of the continuity of the *L*(**r**) function in space, leading to alternating local maxima and minima either along directions or on surfaces. For instance, a (3,−1) CP can be found as a maximum/minimum along a 1D gradient field line of the *L*(**r**) distribution linking two (3,+1) or two (3,−3) CPs. On the other hand, a (3,+1) CP can be found as a maximum/minimum along a 1D gradient field line of the *L*(**r**) distribution linking two (3,+3) or two (3,−1) CPs.

The saddle (3,−1) and (3,+1) CPs correspond to the local 1D depletion/2D concentration and 2D depletion/1D concentration, respectively, along the three main directions of the *L*(**r**) distribution at the CP. Thus, in surfaces including two (3,−3) CPs, a (3,+1) CP appears as a CD site in the valence shell of the atom where the interaction access towards the nucleus is more favourable for a nucleophile, whereas a (3,−1) CP appears in between two (3,−3) CPs along the gradient vector line of the *L*(**r**) distribution linking them. Accordingly, (3,−1) CPs generally perform less well than (3,+1) CPs as electrophilic centres, but this is however modulated by their observed *L*(**r**) values. The quality of (3,−1) CPs as electrophilic centres also depends on the relative positions of the (3,−3) CPs: the more distant from each other they are, the better is the electrophilic quality of the (3,−1) CP. Therefore, if the (3,−3) CPs are separated far enough, the (3,−1) CP marks the interaction access direction towards the nucleus for a nucleophile at the intersection of the perpendicular plane with the gradient field direction of the *L*(**r**) distribution linking the (3,−3) CPs. On the other hand, significant overlap of the electron distributions associated with the two (3,−3) CPs leads us to consider the (3,−1) CP as a nucleophilic indicator of the simultaneous contributions of both (3,−3) CPs, in particular if the former is placed in the intermolecular interaction plane rather than the latter two.

**Figure 2:**
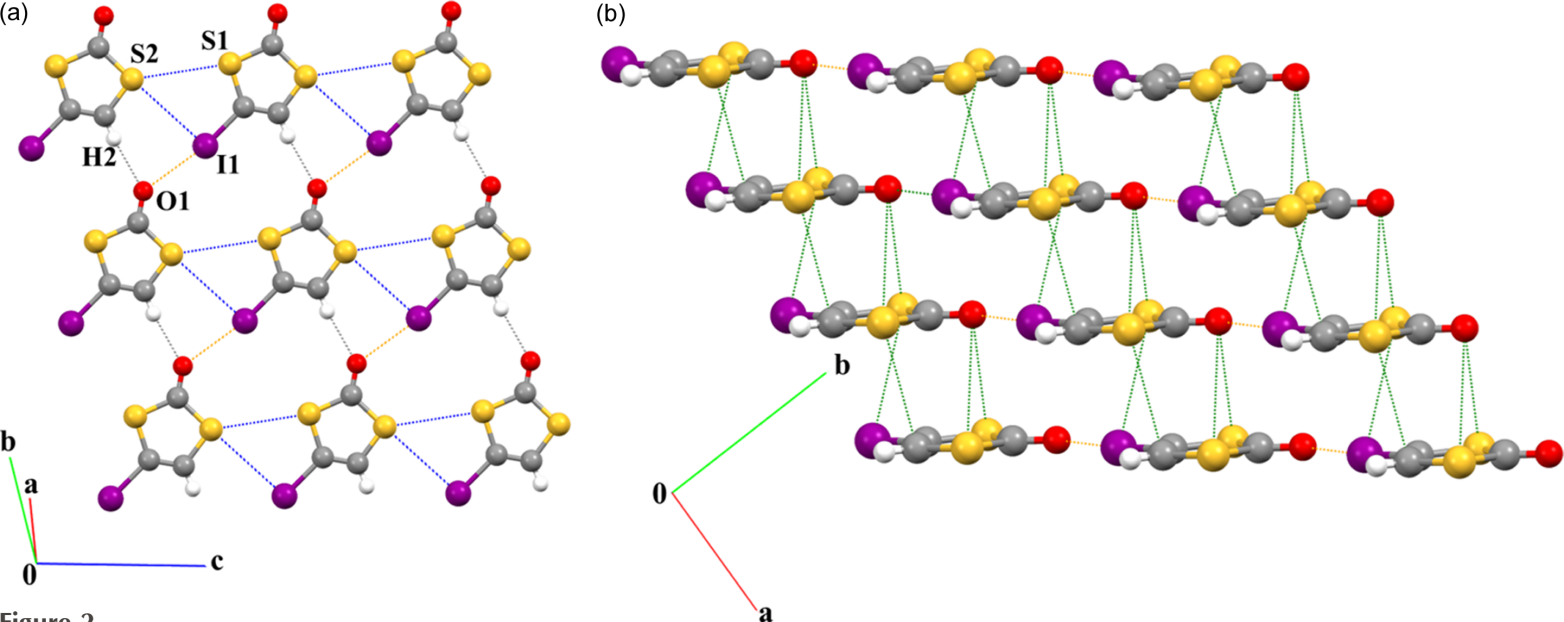
Crystal structure of IDT. (*a*) View from above of layers showing the four-membered fragment 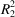
(4) involving two ChB interactions (S⋯S and S⋯I in blue) and a V-type supramolecular motif involving HB (grey) and XB (orange) interactions. (*b*) Side view of layers showing intercolumn contacts in green.

For S_*sp*^3^_ atoms, it is common to observe the lone pairs overlapping each other in the region where a (3,−1) CP is exhibited, whereas for Se_*sp*^3^_ atoms their open valence-shell structure – attributed to large σ-hole regions – results in a (3,−1) CP simultaneously positioned in between two (3,−3) CPs (lone pairs) and two (3,+1) CPs, lying approximately at the intersection of the lone-pair plane and the plane containing both σ holes in a region of significant depletion of electron density. As shown in this work, the last trend is observed for Se_*sp*^3^_ atoms and the (3,−1) CP is considered to be a CD site, while due to the closer proximity of the (3,−3) CPs (lone pairs) of the S_*sp*^3^_ atoms, the (3,−1) CP takes the role of a CC site in IDT because it is placed in the intermolecular interaction plane, representing the accommodation of the contribution of each lone pair that is also involved in another more straightforward interaction [see the interlayer interactions in Fig. 2 ▸(*b*)]. For O_*sp*^3^_ atoms, the overlap of lone pairs is even more significant because they are closer to each other (Brezgunova *et al.*, 2013 ▸). In that case, due to their proximity, the two (3,−3) CPs can contribute as two nucleophilic centres involved in the same intermolecular interaction, without consideration of the less performant (3,−1) CP between them.

## 3. Cambridge Structural Database searches

The Cambridge Structural Database (CSD) *ConQuest* module (version 2021.1.0) was used to perform a search for the intermolecular and non-covalent intramolecular interactions focused on in this study. In addition to several geometrical parameters that have been defined to characterize the interactions, we also applied some constraints for all the searches performed in this study. Hence, only structures whose 3D coordinates are fully determined and error free were involved in the search query. Furthermore, disordered structures, polymers, ionic and powder structures were excluded from the search. Finally, to ensure that only high-quality supramolecular motifs were part of the working set of crystal structures, the agreement *R* factor of the crystal structures, which represents the goodness of fit between the crystallographic model and the experimental diffraction data, was kept at ≤0.1 for all searches.

## 4. Results and discussion

### 4.1. Crystal structure of IDT

IDT crystallizes in the triclinic system (*P*1 space group) with one molecule in the unit cell (see Table S1 for crystallographic data as well as data for spherical and multipolar refinements). In the crystal structure, IDT molecules form layers where the molecules are connected by two types of bifurcated interactions [Fig. 2 ▸(*a*)]. Along the *c*-axis direction, each molecule is connected to neighbouring ones by relatively short S_2_⋯S_1_ and S_2_⋯I_1_ ChB interactions [3.8308 (1) and 3.8035 (9) Å, respectively], forming chains. The value of the reduction ratio (*RR*), which measures the penetration (*RR* < 1) or separation (*RR* > 1) of van der Waals (vdW) spheres, reaches 1.06 and 1.01 for the S_2_⋯S_1_ and S_2_⋯I_1_ ChB interactions, respectively. The molecules are also assembled in the layers by HB (O_1_⋯H_2_) and XB (O_1_⋯I_1_) interactions between the chains described above, with distances [2.235 Å and 2.9342 (3) Å, respectively] that are significantly shorter than the sum of their corresponding vdW radii (*RR* = 0.82 and 0.84). The relatively short distance between the layers (3.339 Å) is a result of the intra-column S_1_⋯I_1_, S_1,2_⋯O_1_ and S_2_⋯C_2_ (lp⋯π) contacts [Fig. 2 ▸(*b*)] between stacked molecules that are slipped within each column.

### 4.2. Chalcogen-bonding interactions

#### 4.2.1. Comparison between chalcogen bonding in IDT and SePA

**Figure 3:**
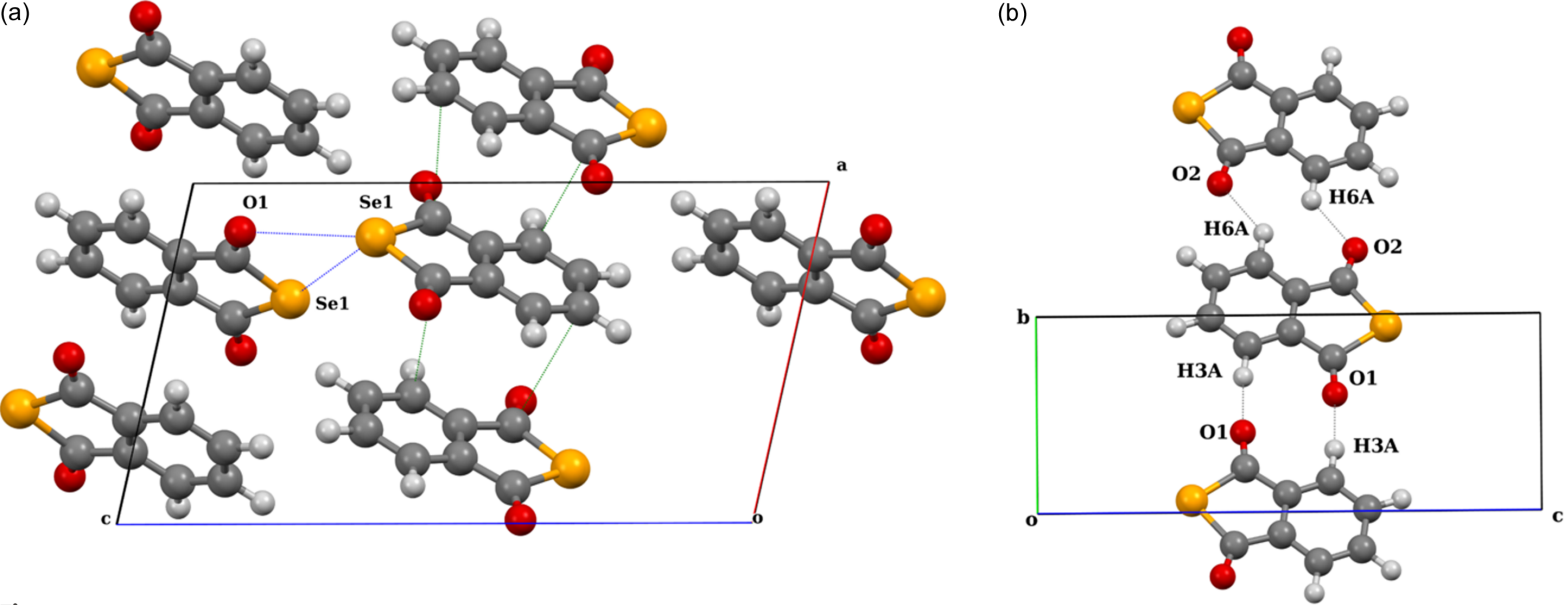
Crystal structure of SePA. (*a*) Side view projected on the *ac* plane showing the four-membered 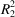
(4) fragment involving two ChB interactions (Se⋯Se and Se⋯O in blue) and intracolumn interactions (green). (*b*) View from above projected on the *bc* plane showing two 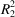
(10) fragments, each involving two HB interactions (grey).

The ChB interactions in the crystal structure of IDT show features very similar to those observed in the crystal structure of SePA (Fig. 3 ▸) (Brezgunova *et al.*, 2013 ▸). Indeed, despite different atoms and relative positions of the interacting molecules, IDT and SePA exhibit similar types of intermolecular contacts involving two Ch_*sp*^3^_ atoms and form a geometrically similar synthon-type fragment that is based on a cyclic four-centre motif. Based on the generalized graph-set notation, the four-membered fragments observed in IDT and SePA (Figs. 2 ▸ and 3 ▸) can be classified as 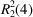 supramolecular motifs, with two electrophilic regions and two nucleophilic regions in the four-membered rings.

**Figure 4:**
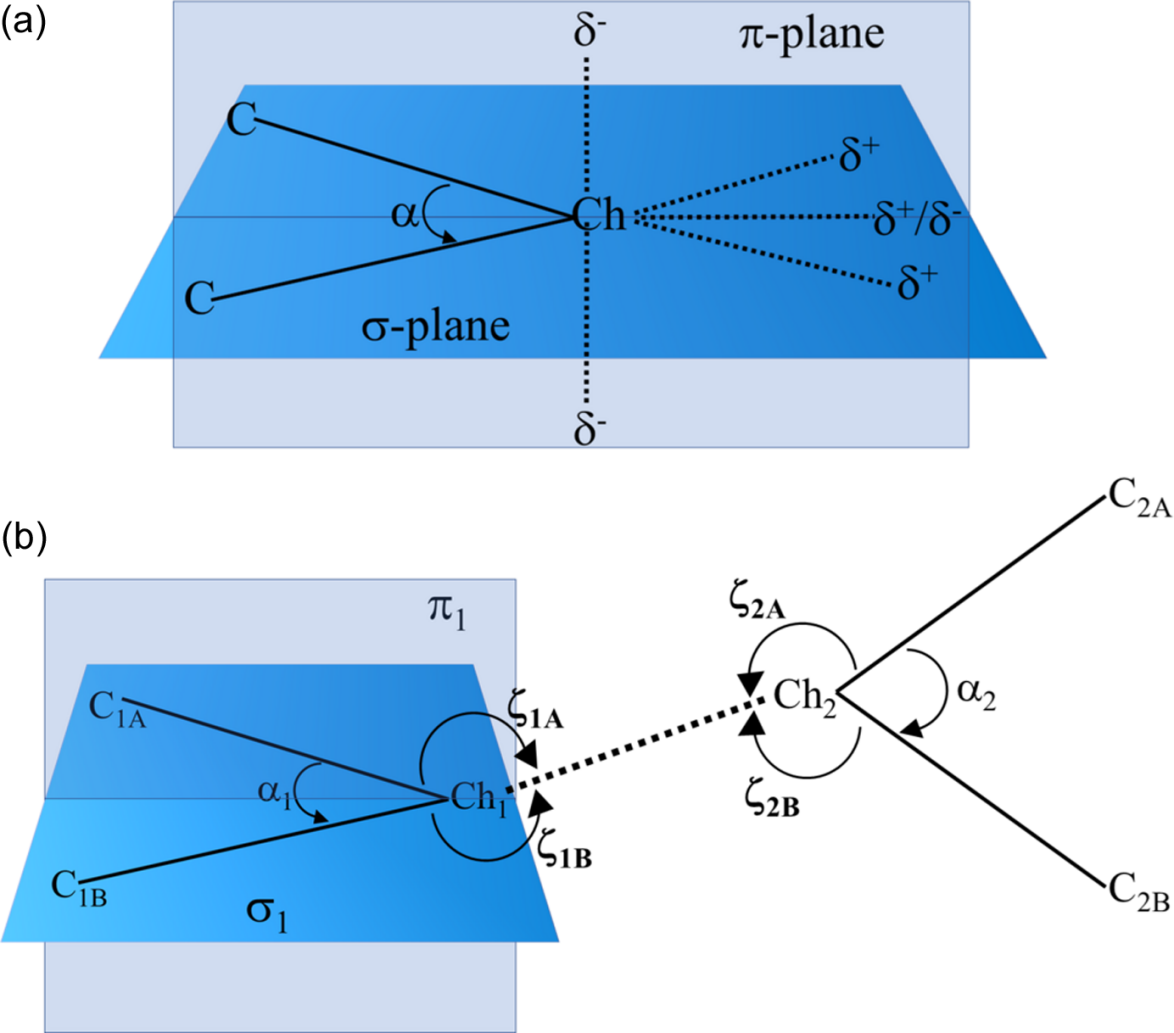
(*a*) Schematic of the expected orientation of electrophilic (δ^+^) and nucleophilic (δ^−^) regions around the Ch atom within the C—Ch—C σ plane and in the perpendicular π plane. (*b*) Structural angles used in the characterization of the C—Ch_1_⋯Ch_2_—C interactions (Ch_1_/Ch_2_ = S/Se/Te).

Table 1 ▸ presents the observed Ch⋯*Y* (*Y* = Ch, *X*, where *X* represents a halogen) distances in IDT and SePA, which are compared with the corresponding sums of the vdW radii by means of the *RR* value, as well as the contact angles ζ_1_ and ζ_2_, indicating the directionality of the interactions (Fig. 4 ▸). From the calculated *RR* parameters, it is observed that Se atoms in the Se⋯Se contact approach closer than the S atoms in the S⋯S contact. This trend has already been noted for halogen atoms, where the heavier bromine atoms approach closer in Br⋯Br contacts than the lighter chlorine atoms in Cl⋯Cl contacts (Brezgunova *et al.*, 2012 ▸).

The structural angles ζ_1_ and ζ_2_ (Table 1 ▸) allow us to roughly estimate the expected locations of δ^+^ and δ^−^ regions in Ch atoms that are involved in the formation of δ^+^⋯δ^−^ interactions (Fig. 4 ▸). The magnitudes of the angles show several differences in the interaction geometries. Thus, for S_1_⋯S_2_, Se_1_⋯Se_1_ and S_2_⋯I_1_ interactions, one of the two ζ_1_ angles tends to be close to 180° (linearity), predicting the location of the δ^+^ region close to the direction of the C—Ch bond. In contrast, for Se_1_⋯O_2_, the ζ_1_ values are close to each other, indicating that the location of the δ^+^ region is expected to be approximately along the direction bisecting the C—Se—C angle in the σ_1_ plane (Fig. 4 ▸), as pointed out in the earlier study of SePA (Brezgunova *et al.*, 2013 ▸). On the other hand, the inverse situation is observed with the ζ_2_ parameter. Indeed, for Se_1_⋯Se_1_, S_2_⋯I_1_ and Se_1_⋯O_2_, ζ_2_ ranges between 90 and 110°, which approximately corresponds to the expected angular location of the lone pairs (δ^−^ regions) in I, O and Se atoms. In the case of the S_1_⋯S_2_ contact, the ζ_2A_ and ζ_2B_ values are close to each other, indicating that the δ^−^ region is roughly located along the direction bis­ecting the C—S_2_—C angle in the σ_2_ plane. It is important to highlight that the observation of a region of either δ^+^ or δ^−^ character along the direction bis­ecting the C—Ch_*sp*^3^_—C angle is the consequence of the angular opening of the Ch_*sp*^3^_ lone pairs. Thus, a δ^−^ region is often observed for lighter chalcogens (O, S), whereas a region of δ^+^ character is more likely to be observed for heavier chalcogens (Se, Te), in which the lone pairs are more separated from each other.

A in-depth structural analysis of chalcogen–chalcogen interactions is needed for the description of geometrical features beyond the standard geometrical contact angles ζ_1_ and ζ_2_, also taking into account the relative orientation of molecules participating in the interactions. This is because not all Ch⋯Ch contacts involve electrophilic⋯nucleophilic (δ^+^⋯δ^−^) ChB interactions, so it is important to differentiate between several cases. For an *sp*^3^-hybridized Ch atom, the σ plane (defined as the C—Ch—C plane) represents the region where the chalcogen atom is expected to develop δ^+^ regions. On the other hand, the π plane, which bis­ects the α(C—Ch—C) angle and is perpendicular to the σ plane, represents the region where the lone pairs of Ch should show participation with δ^−^ regions [Fig. 4 ▸(*a*)]. Accordingly, the ζ_*i*_ and α_*i*_ angles, and the relative position of the σ_*i*_ and π_*i*_ planes of both molecules (*i* = 1, 2) geometrically characterize the relative orientation of the Ch_1_⋯Ch_2_ contact [Fig. 4 ▸(*b*)]. In fact, if Ch_1_ is participating via a δ^+^ region in the C_1A/1B_—Ch_1_⋯Ch_2_ interaction, Ch_2_ should be close to the σ_1_ plane, meaning that the magnitude of ϕ_1_ (defined as ϕ_1_ = α_1_ + ζ_1A_ + ζ_1B_) should approach 360°. Similarly, if Ch_2_ participates via a δ^+^ region in the interaction, then Ch_1_ should be close to the σ_2_ plane and ϕ_2_ ≃ 360°. Other significant geometrical parameters are ζ_1_ = max(ζ_1A_, ζ_1B_) and ζ_2_ = max(ζ_2A_, ζ_2B_), which characterize the directionality of the Ch_1_⋯Ch_2_ interaction. Hence, based on the parameters ϕ_1_, ϕ_2_, ζ_1_ and ζ_2_, chalcogen–chalcogen interactions can be easily differentiated between chalcogen bonding (where regions of different electronic nature δ^+^⋯δ^−^ are facing each other) and chalcogen contacts involving regions of similar electronic nature. The importance of these parameters and their use will be further discussed during the analysis of the CSD search results below.

#### 4.2.2. Topological analysis of ρ(**r**) in chalcogen-bonding regions of *R*_2_^2^(4) motifs in IDT and SePA

**Figure 5:**
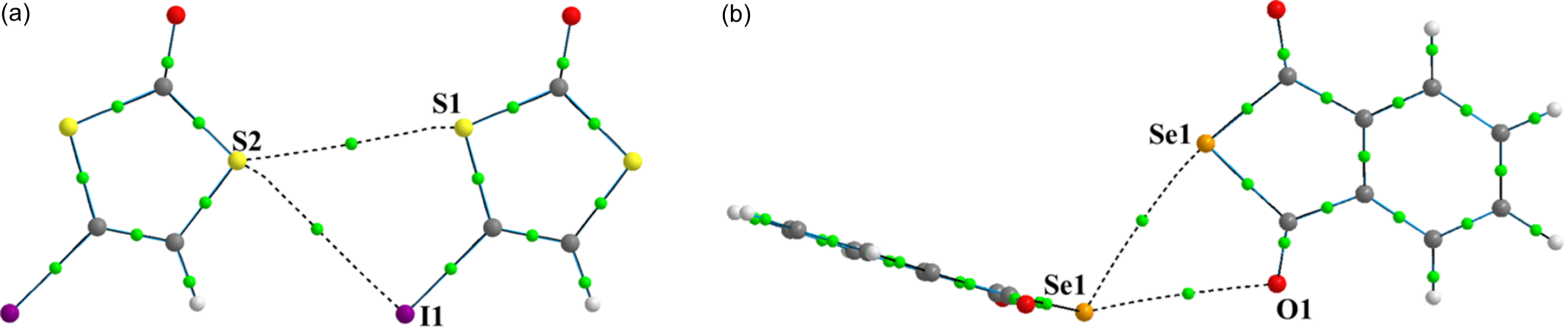
Intermolecular Ch⋯Ch and Ch⋯*Y* contacts (Ch = S, Se; *Y* = I, O) with their bond paths (dotted lines) and their corresponding BCPs (small green circles) between the interacting atoms in the dimers of (*a*) IDT and (*b*) SePA.

Based on the topological analysis of ρ(**r**) (Bader, 1990 ▸), bonding interactions between atoms are identified by the existence of a bond path between their nuclei and the concomitant BCP at the intersection of the bond path with the interatomic zero-flux surface *S*(**r**) [∇ρ(**r**) · **n**(**r**) = 0, ∀**r** ∈ *S*, with **n** being the unit vector ⊥ *S* at **r**]. Chalcogen-bonding interactions in IDT and SePA were thus identified by the observed bond paths and BCPs in their S_1_⋯S_2_, S_2_⋯I_1_, Se_1_⋯Se_1_ and Se_1_⋯O_2_ regions (Fig. 5 ▸).

In order to quantitatively characterize the intermolecular interactions observed in IDT and SePA, we focus on the topological and energetic properties of ρ(**r**) at their BCPs. Table 2 ▸ presents the values of the electron density ρ and the Laplacian *∇*^2^ρ, as well as those of the local electron kinetic (*G*) and potential (*V*) energy densities at the BCPs of the selected interactions. For the sake of comparison between IDT and SePA, as well as for a coherent comparison with data throughout this work, values are reported for interacting dimers calculated at the DFT B3LYP/Def2TZVPP level of theory using experimental geometries (experimental data for SePA, and periodic theoretical *VASP* data for IDT and SePA are presented in Table S2 of supporting information, showing quite similar values). For the four intermolecular interactions, the magnitudes of ρ and ∇^2^ρ are low (0.028 < ρ < 0.050 e Å^−3^, 0.32 < ∇^2^ρ < 0.62 e Å^−5^) (Table 2 ▸) and typically fall into the range determined (theoretically and experimentally) for other weak intermolecular interactions, such as weak H⋯O (Espinosa *et al.*, 1999 ▸), H⋯N (Mata *et al.*, 2007 ▸) and H⋯F (Espinosa *et al.*, 2002 ▸) hydrogen bonds, and weak *X*⋯*X* halogen-bonding interactions (Brezgunova *et al.*, 2012 ▸).

The ratio |*V*|/*G* < 1 classifies the four contacts as ‘pure’ closed-shell interactions of weak intensity (Espinosa *et al.*, 2002 ▸). Although the empirical estimation of the interaction energy *E*_int_ (kJ mol^–1^) = *V* (kJ mol^−1^ bohr^−3^)/2 (Espinosa *et al.*, 1998 ▸) was previously derived for weak-to-medium HB interactions, it has also been used to provide a reasonable estimate for other weak XB (C_6_Br_5_OH, C_6_Cl_5_OH) and ChB (SePA) interactions, which were thus compared in the same framework to HB interactions in the same crystal structures (Brezgunova *et al.*, 2012 ▸; Brezgunova *et al.*, 2013 ▸). In order to gauge the goodness of the estimates for halogen bonds and chalcogen bonds, we have also calculated the *E*_int_ values from other relationships found in the literature and derived for XB and ChB interactions (Bauzá & Frontera, 2020 ▸). Interaction energies in both approaches are similar to each other (except for the S⋯S and S⋯I interactions, which cannot be estimated from the latter reference) and fall within a narrow range of small magnitudes (−*E*_int_ = 2–6 kJ mol^−1^, Table 2 ▸). The addition of individual interaction contributions to estimate the interaction energy of the system in which they are embedded compares very well to the interaction energy of each dimer calculated at the B3LYP-D3/Def2TZVPP level of theory (Table 2 ▸), which exhibits low *E*_int_ magnitudes (∼6 kJ mol^−1^ for IDT and between 8 and 10 kJ mol^−1^ for SePA). Low interaction energies contrast with the formation of similar supramolecular motifs embedded in different crystalline environments, despite the different atoms and functional groups involved.

Comparing the estimated *E*_int_ values derived for interactions involving homochalcogens, the Se⋯Se interaction is roughly 30% more energetic than S⋯S (3.8 kJ mol^−1^ versus 2.7 kJ mol^−1^, as averaged from experimental and theoretical values). The observed tendency, supported by the calculated |*V*|/*G* magnitudes (Table 2 ▸), is in accordance with theoretical studies (Bleiholder *et al.*, 2007 ▸), showing that the strength of these contacts decreases in the order Te⋯Te > Se⋯Se > S⋯S > O⋯O and reflects the tendency discussed earlier for *X*⋯*X* interactions [−*E*_int_ = 4.9 ± 1.2 kJ mol^−1^ for Br⋯Br versus −*E*_int_ = 4.3 ± 1.1 kJ mol^−1^ for Cl⋯Cl (Brezgunova *et al.*, 2012 ▸)]. It is notable that, paralleling the increase of polarizability from chalcogen to halogen atoms (along the same periodic table row), the estimated magnitude *E*_int_ of individual interactions contributing to the interaction energy of motifs increases from S⋯S to Cl⋯Cl and from Se⋯Se to Br⋯Br.

**Figure 6:**
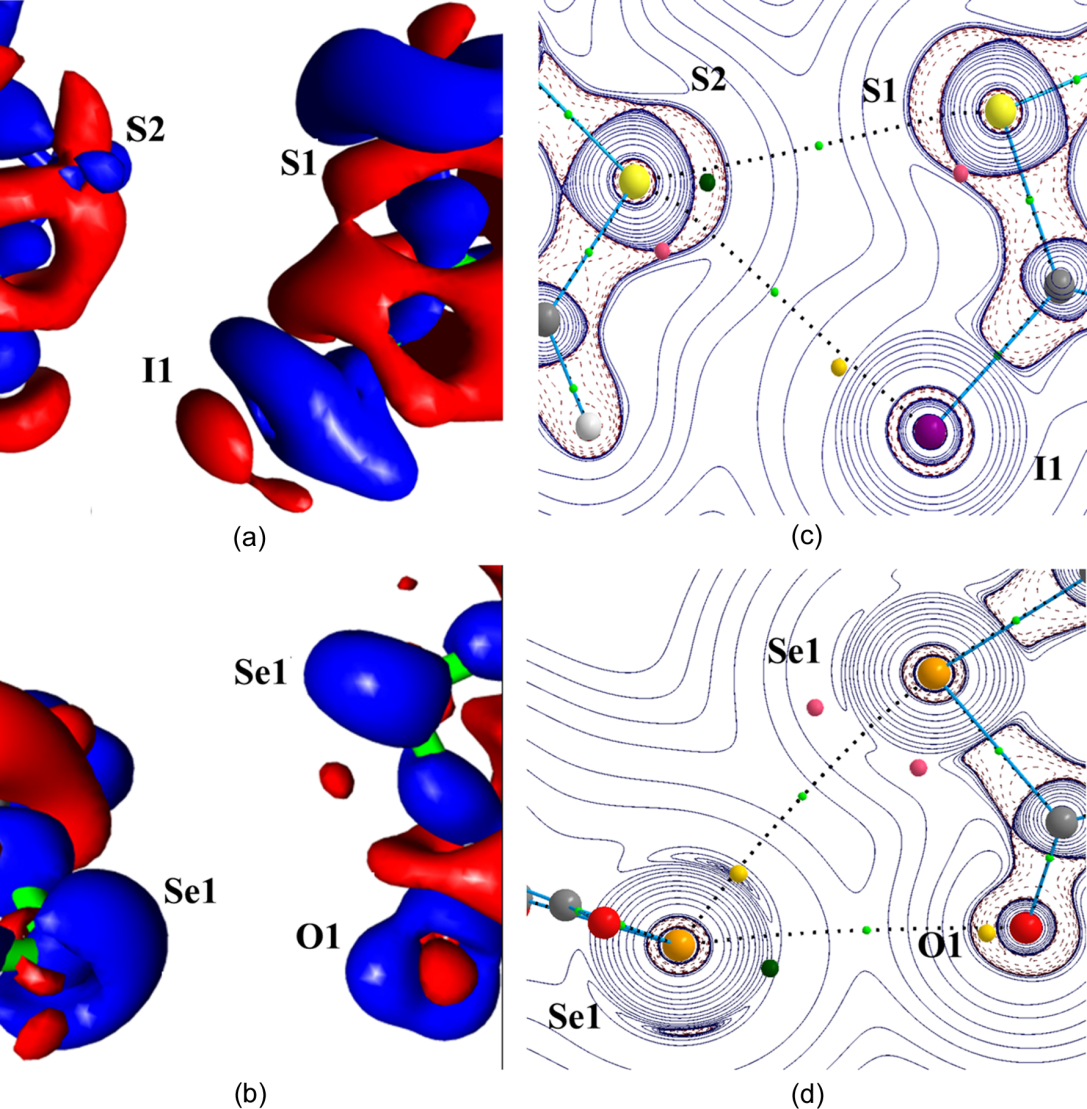
Static deformation density Δρ(**r**) maps in intermolecular regions of (*a*) IDT and (*b*) SePA. Isosurfaces are drawn at ±0.05 e Å^−3^ levels: δ^−^ and δ^+^ isosurfaces are shown in blue and red, respectively. The blue isosurface in front of the S2 atom corresponds to a δ^−^ region in the plane bis­ecting the C1—S2—C2 angle perpendicularly at S2. Maps and relevant topological CPs of the function *L*(**r**) [*L*(**r**) = −∇^2^ρ(**r**)] for (*c*) IDT and (*d*) SePA: CC sites correspond to (3,−3) CPs (yellow) for Se, I and O atoms, and to the (3,−1) CP (green) for S atoms; CD sites correspond to the (3,−1) CP (green) for the Se atom, and to the (3,+1) CPs (pink) for S and Se atoms. All the represented CPs are close to interaction planes. Bond paths (dotted lines) and BCPs (small green circles) associated with the synthon are shown in (*c*) and (*d*) and drawn with the *AIMAll* software (Keith, 2019 ▸).

The deformation of the electron density distribution Δρ(**r**), which is defined as the difference between the crystalline electron density model and the IAM [Δρ(**r**) = ρ_cryst_(**r**) − ρ_IAM_(**r**)], aims to show the redistribution of electrons from isolated atoms to their configuration in the crystal environment as a consequence of all intra- and intermolecular interactions. In Fig. 6 ▸, the theoretical 3D static Δρ(**r**) map is plotted in the chalcogen-bonding regions of IDT and SePA. In both crystal structures, the map clearly shows δ^−^ (Δρ > 0, blue) and δ^+^ (Δρ < 0, red) regions directed towards each other in the intermolecular regions S_2_⋯I_1_ and Se_1_⋯Se_2_, while a less clear situation is observed for the S_1_⋯S_2_ and Se_1_⋯O_2_ contacts involving regions located just in front of the Ch atoms. Paralleling the progressive separation of the lone pairs along the series O < S < Se < Te (Brezgunova *et al.*, 2013 ▸), a δ^−^ region is more likely to be observed with a banana shape in the π plane for O and S atoms, while a δ^+^ region is instead seen with a banana shape in the σ plane from the contributions of the σ holes roughly along the C—Ch directions for Se and Te atoms. The former scenario corresponds to S_1_ in IDT, whereas the polarization of the electron distribution towards one lone pair in S_2_ leads to the splitting of the δ^−^ banana-shape distribution, permitting the emergence of a δ^+^ region [see Fig. 1 ▸(*b*) and insets]. In the plane of the 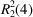 motif [Fig. 6 ▸(*a*)], the S_2_⋯S_1_ ChB interaction (δ^−^⋯δ^+^) involves a depleted region δ^+^ of S1 roughly along the C1—S1 bond (close to the S1—C3 BCP) and a small δ^−^concentration region for S2 (resulting after splitting) close to the intersection of the σ and π planes (in the vicinity of the δ^−^ banana-shape distribution). In SePA, the Se atom also exhibits a banana-shape distribution in the π plane [Fig. 6 ▸(*b*)], but due to the increased separation of the lone pairs with respect to S atoms, a small δ^+^ region appears in the σ plane, relatively close to the δ^−^ distribution in the π plane.

In order to clarify these observations, the interactions that are focused on have been further characterized using the topological CPs of the function *L*(**r**) [Figs. 6 ▸(*c*) and 6 ▸(*d*)]. In the valence shells of atoms, (3,−3) CPs lying outside covalent bonds indicate 3D CC sites and are associated with lone pairs, whereas (3,+1) CPs are mostly found on (or close to) intermolecular interaction planes (along the C—*X* bonding directions for halogen atoms, and in the σ plane roughly along the C—Ch_*sp*^3^_ bonding directions for chalcogen atoms). The (3,+1) CPs are typically associated with CD sites, because the function *L*(**r**) is depleted along two main directions on surfaces where (3,−3) CPs remain. On the other hand, topological CPs of the (3,−1) type indicate that the function *L*(**r**) is depleted along only one main direction that links two (3,−3) CPs, also as a consequence of the continuity of the function in space. Accordingly, close to the π plane of Ch_*sp*^3^_ atoms, a (3,−1) CP is exhibited between two (3,−3) CPs that correspond to the CC sites of the lone pairs. Note that the separation between the two (3,−3) CPs and the sign of the function *L*(**r**) in the region of the (3,−1) CP are irrelevant for the topological emergence of this CP. Therefore, (3,−1) CPs can show as either weak CC or weak CD sites because this feature only depends on the separation of the (3,−3) CPs, which are associated with the relative position of the lone pairs and therefore to the contribution of a small region of either δ^−^ or the δ^+^ character in the vicinity of the intersection of the σ and π planes. Hence, the (3,−1) CP of the S2 atom in Fig. 6 ▸(*c*) represents a CC site, whereas in the case of the Se1 atom in Fig. 6 ▸(*d*), it represents a CD site. Accordingly, in both crystal structures, the synthon shows facing CD⋯CC interactions, while aligning the corresponding electrophilic⋯nucleophilic interactions with internuclear directions, and are observed close to bond paths. Indeed, the angle between the directions α(CD_1_⋯CC_2_; at_1_⋯at_2_) is small (10.1, 8.7, 11.7 and 17.3° for S⋯S, S⋯I, Se⋯O and Se⋯Se, respectively), as previously observed in another crystal structure involving ChB interactions (Shukla *et al.*, 2020 ▸). Note that, while a high similarity is observed between the positions of CC and CD sites within the same motif found in SePA and IDT, due to the extended δ^+^ region around the Se atom in the molecular σ plane a further (3,+1) CP is also observed on the other side of the bond path [upper position in Fig. 6 ▸(*d*)]. This CP remains roughly along the same C–Se direction and belongs to the same σ-hole region, with α = 15.0°. Accordingly, the CC site seems to interact simultaneously with two CD sites of the same Se atom, with α = 17.3 and 15°. This simultaneous interaction of one CC site with two CD sites observed on either side of the same bond path is perhaps at the origin of slightly higher α values than those typically observed with all the other interactions found in this work (<15°), as a consequence of the accommodation of two CD⋯CC interactions. Altogether, the reported results support the assumption that the relative orientation of atoms involved in intermolecular interactions, and therefore the supramolecular motifs they form, are driven by the local electrophilic and nucleophilic regions found in the valence shells of atoms, which are governed by specific CD and CC sites determined by the topological CPs of the function *L*(**r**).

#### 4.2.3. CSD search on *R*_2_^2^(4) supramolecular motifs

**Figure 7:**
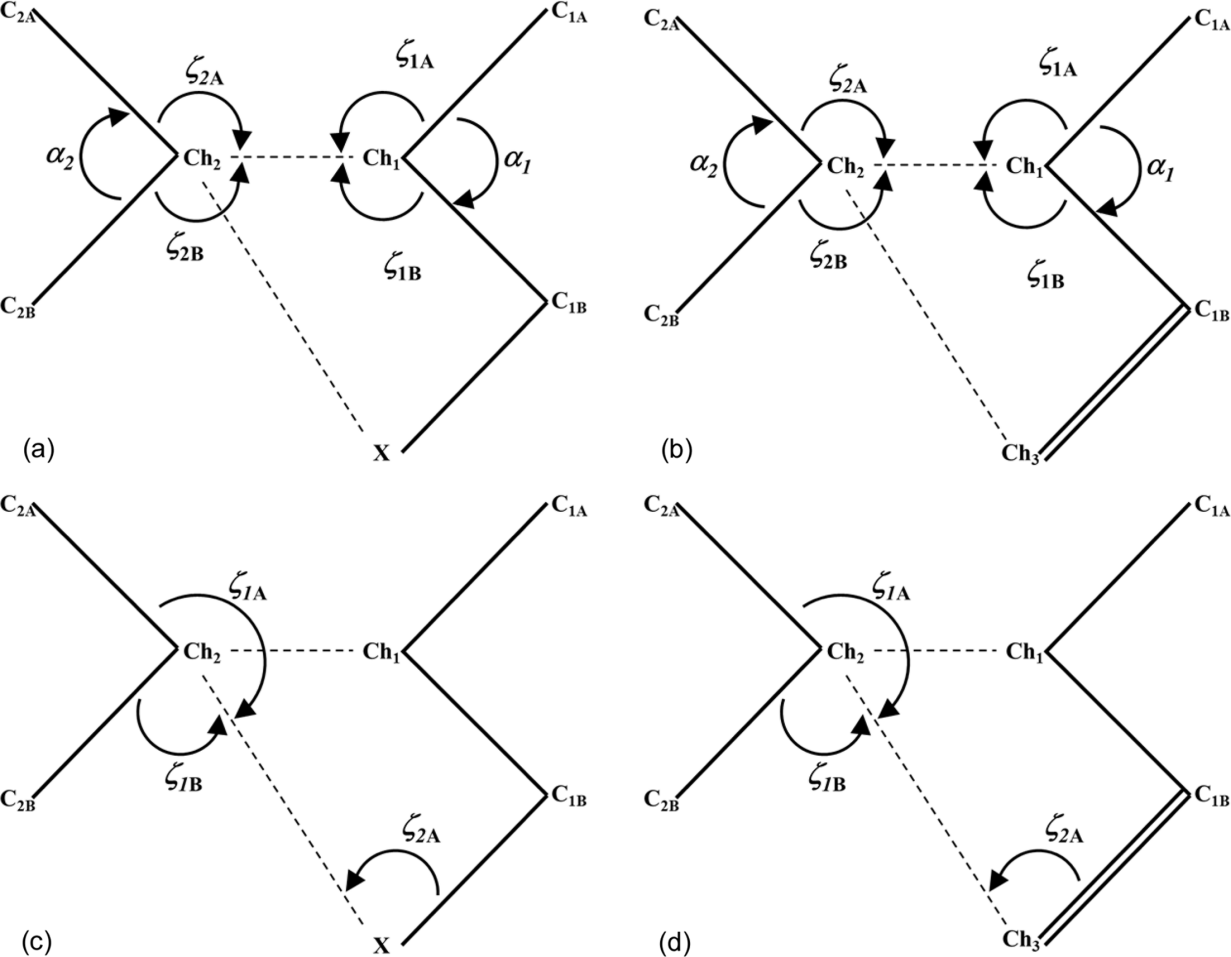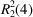 supramolecular motifs used for the CSD search: Ch_1_/Ch_2_ = S/Se/Te, *X* = F/Cl/Br/I, Ch_3_ = O/S/Se (no fragments were found with Ch_3_ = Te). Geometrical criteria used in the search for motifs: distances *d* = Ch_1_⋯Ch_2_/Ch_2_⋯*X*/Ch_2_⋯Ch_3_ ≤ sum (vdW) + 0.4 Å, structural angles ∠C—Ch_1_⋯Ch_2_/∠C—Ch_2_⋯Ch_1_/∠C—Ch_2_⋯*X*/∠C—*X*⋯Ch_2_/∠C—Ch_2_⋯Ch_3_/∠C=Ch_3_⋯Ch_2_ within the range 60–180°. For C_1A/1B_—Ch_1_⋯Ch_2_—C_2A/2B_ interactions: ζ_1A/1B_ = ∠C_1A/1B_—Ch_1_⋯Ch_2_ and ζ_2A/2B_ = ∠C_2A/2B_—Ch_2_⋯Ch_1_. For C_2A/2B_—Ch_2_⋯*X*/Ch_3_—C_1B_ interactions: ζ_1A/1B_ = ∠C_2A/2B_—Ch_2_⋯*X* and ζ_2_ = ∠C_1B_—*X*/Ch_3_⋯Ch_2_. Note that, in addition to Ch_1_⋯Ch_2_ interactions, the nomenclature ζ_1A/1B_ and ζ_2A/2B_ also requires Ch_2_⋯*X* interactions to be consistent with that of Table 1 ▸. *X*-atom vdW radii = 1.47, 1.75, 1.85 and 1.98 Å for F, Cl, Br and I, respectively. Ch-atom vdW radii = 1.52, 1.80 and 1.90 Å for O, S and Se, respectively.

**Figure 8:**
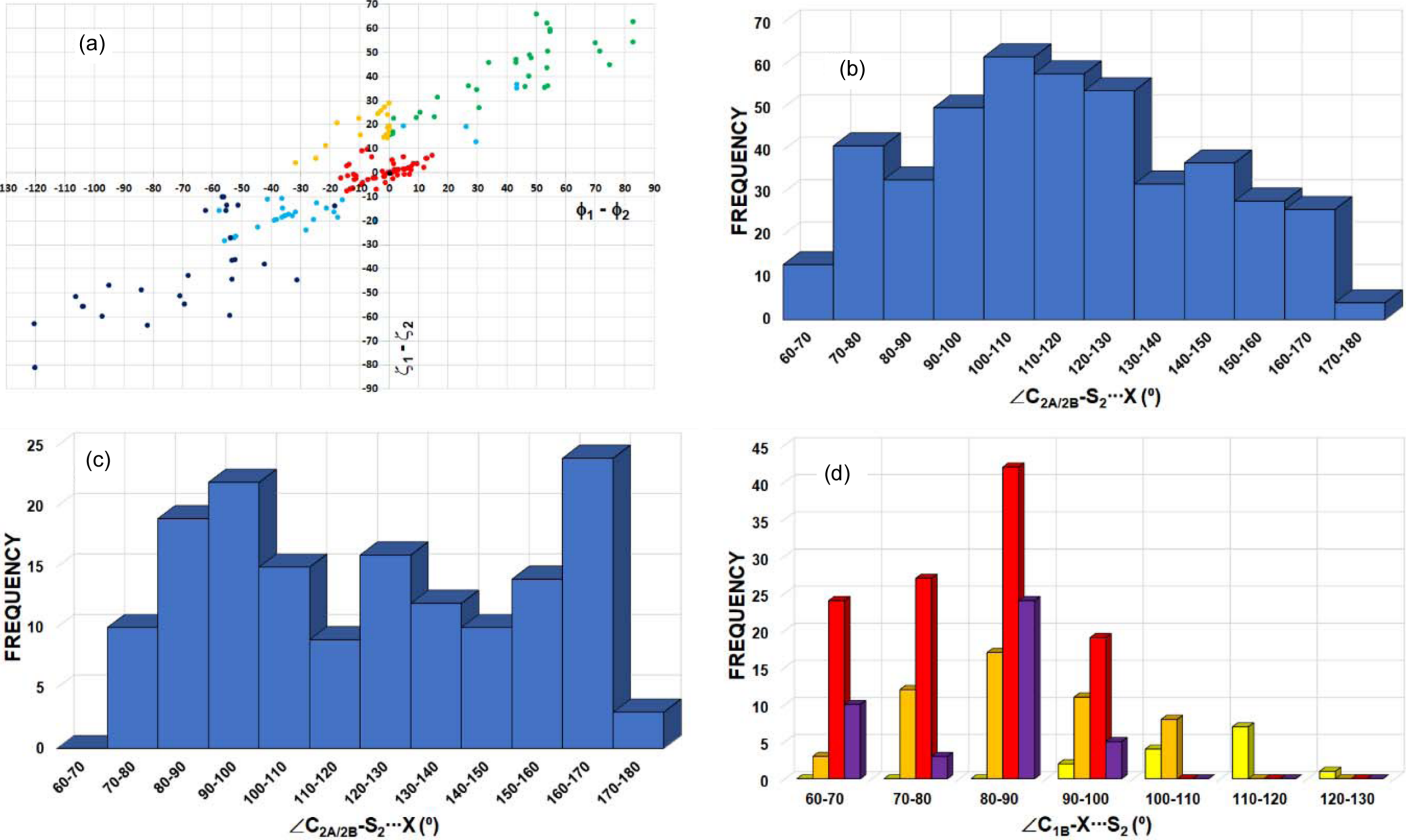
(*a*) Scatter plot of ζ_1_ − ζ_2_ versus ϕ_1_ − ϕ_2_ with Ch_1_ = Ch_2_ = S and *X* = F/Cl/Br/I [Figs. 7 ▸(*a*) and 7 ▸(*c*)], see text for colour code. Frequency plots of geometrical angles: (*b*) ζ_1A/1B_ = ∠C_2A/2B_—S_2_⋯*X* defined in Fig. 7 ▸(*c*) for the whole set of 219 motifs, (*c*) ζ_1A/1B_ = ∠C_2A/2B_—S_2_⋯*X* defined in Fig. 7 ▸(*c*) for the 77 motifs showing Ch_1_⋯Ch_2_ ChB interactions, (*d*) ζ_2A_ = ∠C_1B_—*X*⋯S_2_ (*X* = F/Cl/Br/I) defined in Fig. 7 ▸(*c*) with *X* = F (yellow), *X* = Cl (orange), *X* = Br (red) and *X* = I (purple).

Fig. 7 ▸ shows the geometrical parameters and criteria used for the CSD search. Results indicate that the 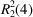 supramolecular motif is primarily present with the sulfur atom in the Ch_1_ and Ch_2_ positions (Ch_1_ = S_1_ and Ch_2_ = S_2_) with either a halogen atom (*X* = F/Cl/Br/I) (219 unique motifs) [Figs. 7 ▸(*a*) and 7 ▸(*c*)] or a chalcogen atom (Ch_3_ = O/S/Se) (513 unique motifs) [Figs. 7 ▸(*b*) and 7 ▸(*d*)]. Focusing on the S_1_⋯S_2_ interaction with *X* in the 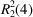 motif [Fig. 8 ▸(*a*)], 57 hits present ϕ_1_ = ϕ_2_ and ζ_1_ = ζ_2_. These motifs are chalcogen⋯chalcogen contacts similar to those previously reported as halogen⋯halogen type-I contacts (Desiraju & Parthasarathy, 1989 ▸). These motifs (set 1) are all located exactly at the origin of the ϕ_1_ − ϕ_2_ versus ζ_1_ − ζ_2_ plot (represented in black). In addition, 54 motifs (set 2) with −20° < ϕ_1_ − ϕ_2_ < 20° and −10° < ζ_1_ − ζ_2_ < 10° (represented in red) can be also considered as type-I contacts, because they show both similar directionality (ζ_1_ ≃ ζ_2_) and relative situation of the S_1_ and S_2_ atoms with respect to the σ_1_ and σ_2_ planes (ϕ_1_ ≃ ϕ_2_). A further 31 motifs (set 3) show both ϕ_1_ and ϕ_2_ < 320° (represented in light blue). The significant difference of ϕ_1_ and ϕ_2_ with respect to 360° implies that neither S_2_ nor S_1_ is close to the σ plane of the other S atom, indicating that their δ^+^ regions are not involved in their contacts and therefore suggesting that a primarily molecular stacking builds the motifs. The remaining 77 motifs show typical behaviour for chalcogen bonds involving electrophilic and nucleophilic regions in the interaction (δ^+^⋯δ^−^). In 31 of these motifs (set 4), ϕ_1_ > ϕ_2_ (ϕ_1_ − ϕ_2_ > 0) and ζ_1_ > ζ_2_ (ζ_1_ − ζ_2_ > 0) (represented in green) are observed. A positive magnitude of ϕ_1_ − ϕ_2_ implies that S_2_ is in the σ_1_ plane of S_1_, while a positive magnitude of ζ_1_ − ζ_2_ implies that S_1_ is participating via a δ^+^ site, therefore indicating an S_1_^δ+^⋯S_2_^δ−^ interaction. For 27 motifs (set 5), both ϕ_1_ − ϕ_2_ and ζ_1_ − ζ_2_ are negative (represented in dark blue) and they correspond to S_1_^δ−^⋯S_2_^δ+^ interactions by invoking similar reasons. In the remaining 19 motifs (set 6),* ϕ*_1_ − ϕ_2_ < 0 and ζ_1_ − ζ_2_ > 0 (represented in orange). Here, while the positive ζ_1_ − ζ_2_ value means that S_1_ is participating in the interaction with a δ^+^ region, a negative ϕ_1_ − ϕ_2_ value implies that S_1_ is in the σ_2_ plane of S_2_. This is the case for IDT (ϕ_1_ − ϕ_2_ < −3.3° and ζ_1_ − ζ_2_ > 26.0°, Table 1 ▸), which corresponds to a ChB situation where S_2_ is participating with a δ^−^ region roughly located in the vicinity of its σ_2_ plane, along the direction bis­ecting the C_1A_—S_2_—C_1B_ angle, and influenced by the contribution of one lone pair that is polarized in this region.

**Figure 9:**
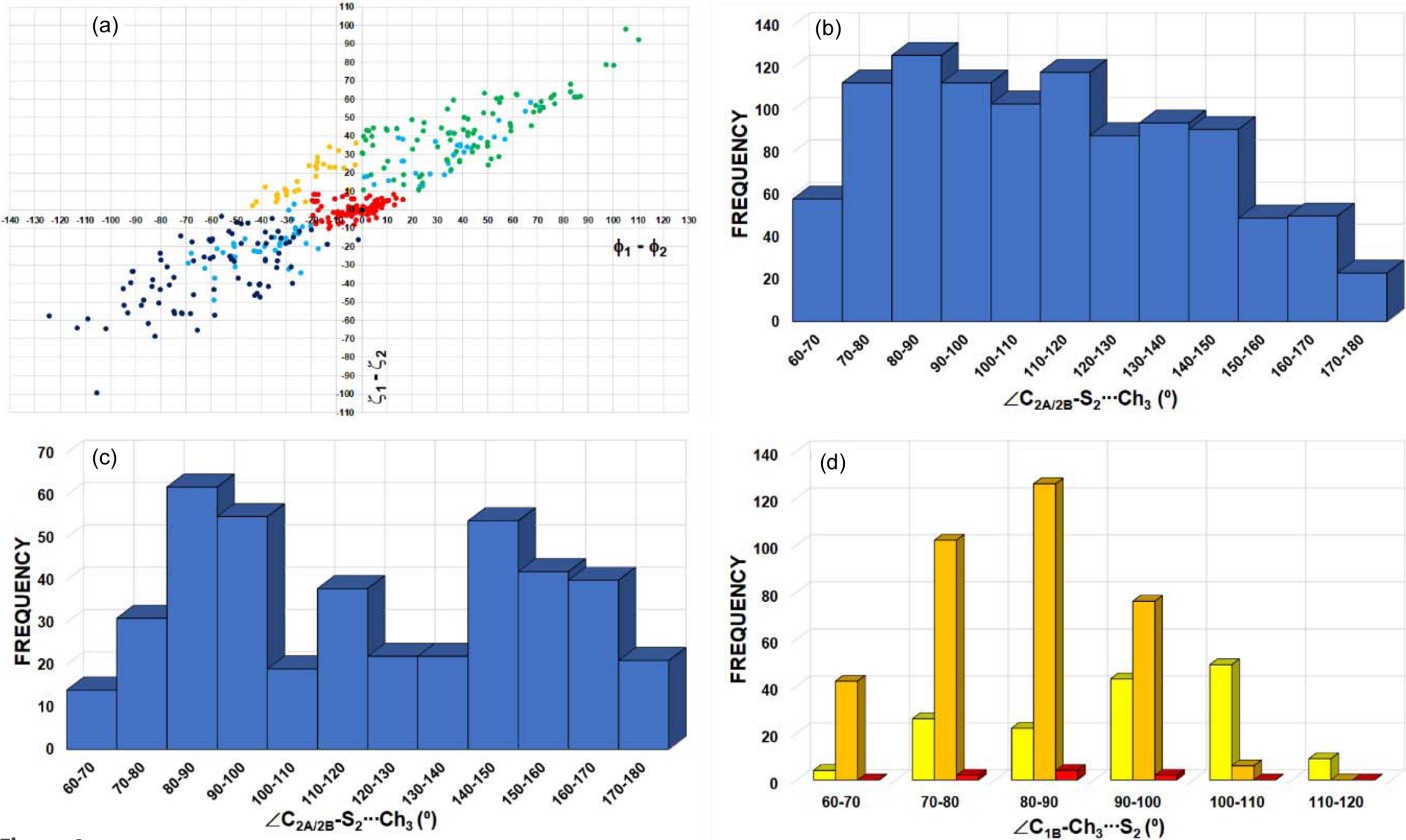
(*a*) Scatter plot of ζ_1_ − ζ_2_ versus ϕ_1_ − ϕ_2_ with Ch_1_ = Ch_2_ = S and Ch_3_ = O/S/Se [Figs. 7 ▸(*b*) and 7 ▸(*d*)] (no fragments were observed with Ch_3_ = Te), see text for colour code. Frequency plots of geometrical angles: (*b*) ζ_1A/1B_ = ∠C_2A/2B_—S_2_⋯Ch_3_ defined in Fig. 7 ▸(*d*) for the whole set of 513 motifs, (*c*) ζ_1A/1B_ = ∠C_2A/2B_—S_2_⋯Ch_3_ defined in Fig. 7 ▸(*d*) for the 210 motifs showing Ch1⋯Ch2 ChB interactions, (*d*) ζ_2A_ = ∠C_1B_—Ch_3_⋯S_2_ (Ch_3_ = O/S/Se) defined in Fig. 7 ▸(*d*) with Ch_3_ = O (yellow), Ch_3_ = S (orange) and Ch_3_ = Se (red) (no fragments were observed with Ch_3_ = Te).

In order to extend the scope of our analysis, we also performed a detailed CSD search for motifs represented in Figs. 7 ▸(*b*) and 7 ▸(*d*), with Ch_1_ = Ch_2_ = S and Ch_3_ = O/S/Se [Fig. 9 ▸(*a*)]. The search resulted in 513 unique motifs that were analysed similarly to Fig. 8 ▸(*a*). In terms of the previously defined subsets of data, the analysis shows the presence of 130 motifs belonging to set 1 (black data, all of them placed at the origin of the graph), 104 to set 2 (red data), 69 to set 3 (light blue data), 91 to set 4 (green data), 87 to set 5 (dark blue data) and 32 to set 6 (orange data), leading to a data distribution which is very similar to that depicted in Fig. 8 ▸(*a*). Also, as in Fig. 8 ▸(*a*), sets 4–6 in Fig. 9 ▸(*a*) represent the motifs formed with Ch_1_⋯Ch_2_ ChB interactions, while sets 1–3 concern type-I and stacking contacts. Note that searching with Ch_1_/Ch_2_ = S/Se/Te (excluding Ch_1_ = Ch_2_ = S) and either *X* = F/Cl/Br/I or Ch_3_ = O/S/Se/Te resulted in very few hits (only 43 and 8 unique motifs, respectively) and hence no in-depth analysis was performed for this dataset, as it was too small.

Overall, the observations made here with respect to S⋯S ChB interactions and contacts are supported by the geometrical parameters ϕ_1_ − ϕ_2_ and ζ_1_ − ζ_2_ (Fig. 8 ▸). Hence, while sets 1 to 3 correspond to S⋯S contacts of type I (roughly |ϕ_1_ − ϕ_2_| < 20° and |ζ_1_ − ζ_2_| < 10°, red data) and stacking geometries (mainly with 15° < |ϕ_1_ − ϕ_2_| < 60° and |ζ_1_ − ζ_2_| > 30°, light blue data, even if some data points can spread out of these limits), sets 4 and 5 are associated with ChB δ^+^⋯δ^–^ interactions (either both ϕ_1_ − ϕ_2_ and ζ_1_ − ζ_2_ are positive or negative, green or dark blue data). The particular S⋯S chalcogen contacts in set 6 (ϕ_1_ − ϕ_2_ < 0 and ζ_1_ − ζ_2_ > 0, orange data) correspond to δ_2_^−^⋯δ_1_^+^ ChB interactions. However, they do not have counterpart δ_2_^+^⋯δ_1_^−^ ChB interactions in the region where ϕ_1_ − ϕ_2_ > 0 and ζ_1_ − ζ_2_ < 0 due to the presence of the Ch_2_⋯*X* contacts, which involve the corresponding electrophilic region of Ch_2_ and the nucleophilic region of *X* in the δ_2_^+^⋯δ*_X_*^−^ ChB interaction, as observed in IDT.

The high similarity between Figs. 8 ▸(*a*) and 9 ▸(*a*) seems to indicate that the structural features involved in the 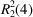 motifs that were analysed are equivalent when either halogen or chalcogen atoms are present at the analogous position (*X* or Ch_3_) in the 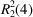 motif. Despite this significant trend, plotting the frequency of the C_2A_/C_2B_—S_2_⋯*X* [Fig. 8 ▸(*b*)] and C_2A_/C_2B_—S_2_⋯Ch_3_ [Fig. 9 ▸(*b*)] angles for all 219 and 513 motifs, respectively, observed for each dataset does not reveal any particular angular preference. However, selecting the 77 and 210 motifs that we consider to be ChB interactions within each dataset, a clear pattern appears in both frequency plots. Two peaks are found in the 90–100° and 160–170° regions for 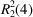 motifs with *X* [Fig. 8 ▸(*c*)] and two others apear in the 80–90° and 140–150° regions for 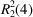 motifs with Ch_3_ [Fig. 9 ▸(*c*)], which correspond to the expected angular geometries of C_2B_—S_2_⋯*X*/Ch_3_ and C_2A_—S_2_⋯*X*/Ch_3_, respectively, associated with the δ^+^ region of the S atom along the C_2A_—S_2_ direction. These peaks in frequency are similar to those previously reported for a CSD search on S/Se/Te⋯O ChB interactions (Brezgunova *et al.*, 2013 ▸). Furthermore, a third lower-frequency peak appears in the ranges 120–130°/110–120° [Figs. 8 ▸(*c*) and 9 ▸(*c*)] for each dataset, which mainly corresponds to a δ^+^ region (smaller than the δ^+^ regions that are approximately along C—S bonding directions) placed in the σ plane along the direction bis­ecting the C_2A_—S_2_—C_2B_ angle and making a ChB interaction with *X*/Ch_3_. In this case, we have not, however, excluded the possibility of finding motifs where a δ^−^ region appears to be involved instead of a δ^+^ region, making a type-I interaction between S and *X*/Ch_3_ atoms, even if this situation should be, in principle, less favourable for the stability of the motif. Figs. 8 ▸(*d*) and 9 ▸(*d*) show the frequency plots for the C_1B_—*X*/Ch_3_⋯S_2_ angles [Figs. 7 ▸(*c*) and 7 ▸(*d*)], depicted separately for each *X* or Ch_3_ atom type. They point the expected angular geometry at which the lone pairs of the *X*/Ch_3_ atoms are involved in the interaction. Accordingly, while the peak of the frequency plot is observed at 80–90° for *X* = Cl, Br and I, and for Ch_3_ = S and Se, the peak shifts towards 110–120° for *X* = F and to 100–110° for Ch_3_ = O.

To conclude, the geometrical analysis above shows that the S⋯S ChB interaction in IDT belongs to set 6 of the CSD search results (ϕ_1_ − ϕ_2_ < 0 and ζ_1_ − ζ_2_ > 0), whereas the Se⋯Se ChB interaction in SePA belongs to set 1 (ϕ_1_ − ϕ_2_ > 0 and ζ_1_ − ζ_2_ > 0) (Table 1 ▸). Both occur simultaneously with a further ChB interaction, involving either a halogen or chalcogen atom as the acceptor. Overall, both ChB interactions build a supramolecular 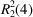 motif, the geometry of which fits with the expected positions of the electrophilic and nucleophilic regions of the atoms in the interaction. Additionally, the electronic descriptors that characterize the electrophilic (CD) and nucleophilic (CC) sites in the valence shells of the atoms are observed in a CD⋯CC interaction within the supramolecular 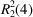 motif, facing each other closely along the internuclear directions. These trends suggest that the formation and the geometry of the synthon in the crystal structures of IDT and SePA are exhibited regardless of the molecule and are highly dependent on the appropriate orientation of CC and CD sites present in the motif. Hence, with the aim of extending this investigation, hereafter we analyse other supramolecular motifs involving different intermolecular and non-covalent intramolecular interactions.

### 4.3. Other synthons and motifs

#### 4.3.1. Triangular *X*_3_ synthons [*R*_3_^3^(3) motifs]

**Figure 10:**
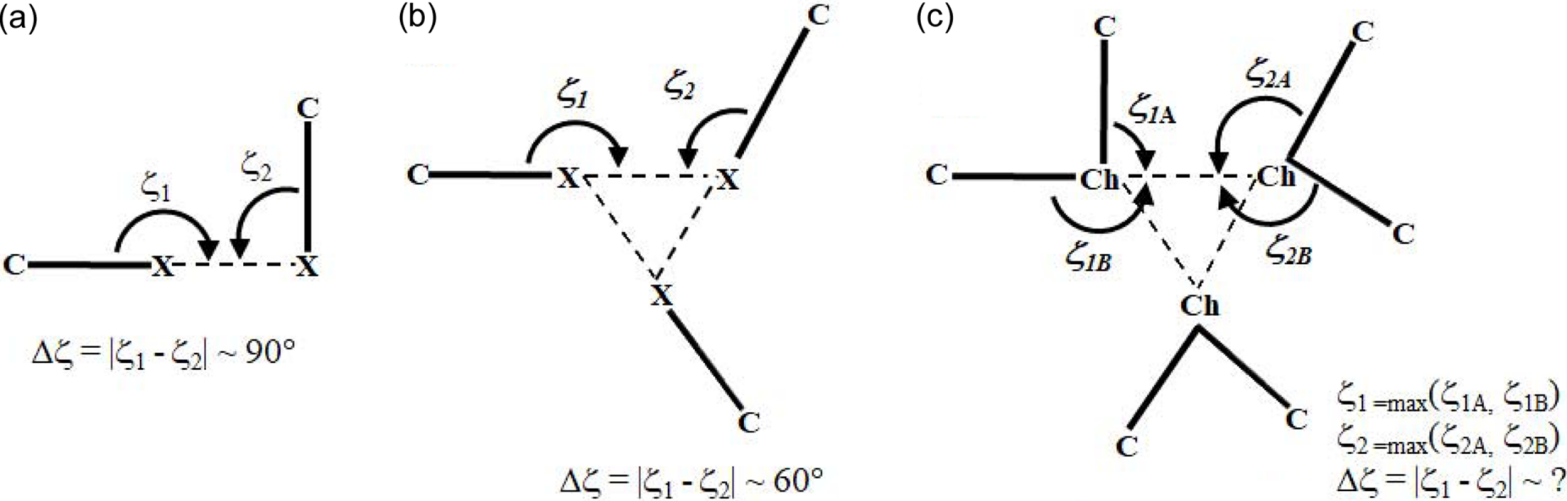
Geometrical representation of *X*⋯*X* halogen bonds (*X* = Cl/Br/I) (*a*) for a single interaction (ζ_1_ ≃ 180°, ζ_2_ ≃ 90°) and (*b*) within a triangular *X*_3_ synthon (ζ_1_ ≃ 180°, ζ_2_ ≃ 120°). (*c*) Ch⋯Ch chalcogen bonds (Ch = S/Se/Te) shown within a triangular Ch_3_ synthon. Δζ is expected at roughly 90 and 60° in (*a*) and (*b*), whereas in (*c*) it is difficult to estimate due to the presence of multiple δ^+^/δ^−^ sites in Ch_*sp*^3^_ atoms [see Fig. 4 ▸(*a*)]. CSD searches for *X*_3_ and Ch_3_ synthons [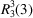 motifs] involve the following criteria: distances *X*⋯*X* and Ch⋯Ch ≤ sum (vdW) + 0.4 Å, and angles ∠C—*X*⋯*X* and ∠C—Ch⋯Ch within the ranges 100–180° and 60–180°, respectively. *X*-atom vdW radii = 1.75, 1.85 and 1.98 Å for Cl, Br and I, respectively. Ch-atom vdW radii = 1.80, 1.90 and 2.06 Å for S, Se and Te, respectively.

Previous studies on the crystal structures of the halogenated molecules hexa­chloro­benzene, C_6_Cl_6_ (HCB), hexa­bromo­benzene, C_6_Br_6_ (HBB), penta­chloro­phenol, C_6_Cl_5_OH (PCP), and penta­bromo­phenol, C_6_Br_5_OH (PBP), have shown the formation of triangular *X*_3_ synthons (Bui *et al.*, 2009 ▸; Brezgunova *et al.*, 2012 ▸; Aubert *et al.*, 2011 ▸), which can be labelled as 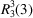 ring motifs following the new graph-set notation, where each halogen atom simultaneously plays an amphoteric role involving one electrophilic and one nucleophilic region in the *X*_3_ synthon. The bonding pattern in these motifs was established to be driven by local electrostatic electrophilic⋯nucleophilic (δ^+^⋯δ^–^) interactions. In terms of geometry, the main difference between a standard single halogen⋯halogen bond and a triangular XB interaction in the *X*_3_ synthon is the relative orientation of the electrophilic (δ^+^) and nucleophilic (δ^−^) regions involved in the formation of the halogen bonds. Thus, in a standard single halogen⋯halogen bond, δ^+^ and δ^−^ sites are roughly orthogonal to each other, resulting in an angular difference |Δζ| = |ζ_1_ − ζ_2_| ≃ 90° [Fig. 10 ▸(*a*)]. On the other hand, in the case of *X*_3_ synthons, the angular difference |Δζ| is significantly less and ideally close to 60° in order to simultaneously accommodate three electrophilic (δ^+^) and nucleophilic (δ^−^) regions [Fig. 10 ▸(*b*)].

Table 3 ▸ presents the geometrical parameters of the *X*⋯*X* interactions within *X*_3_ synthons in C_6_Cl_6_, C_6_Cl_5_OH, C_6_Br_6_ and C_6_Br_5_OH. The *RR* parameter ranges from 0.95 to 1.05, indicating intermolecular distances between the interacting atoms that are close to the sums of their vdW radii. In addition, the magnitude of |Δζ|, which ranges from 47.9 to 59.1° in all but two interactions in C_6_Cl_5_OH, further confirms the characteristic XB interactions δ^+^⋯δ^−^ under investigation. The two interactions that lie out of the expected |Δζ| range in C_6_Cl_5_OH concern a specific molecular orientation, different to those found in the other compounds, which is due to a particular competition between XB and HB interactions.

**Figure 11:**
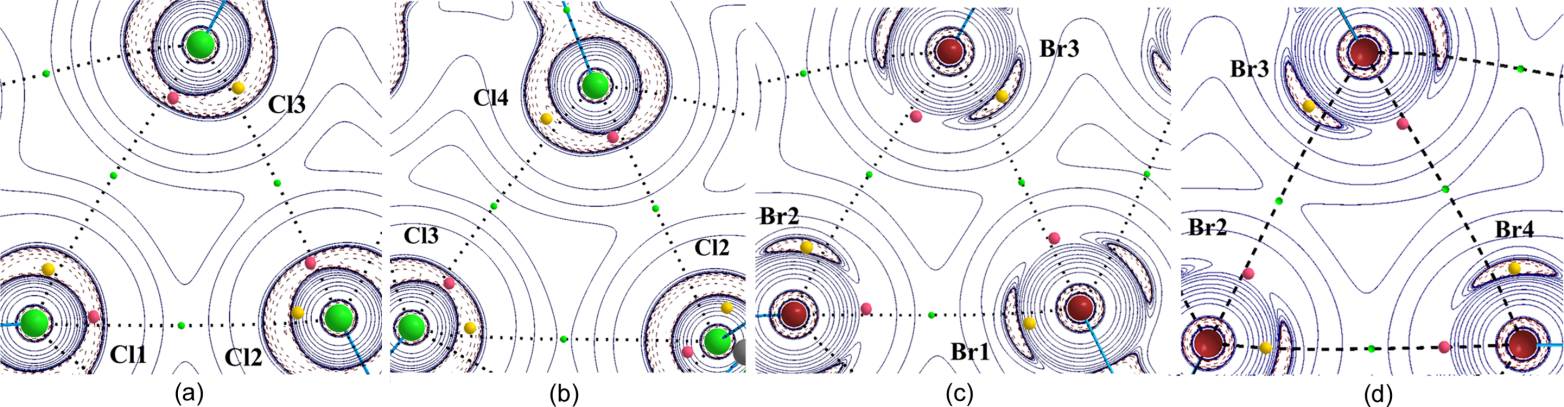
Maps and topological CPs of the function *L*(**r**) [= −∇^2^ρ(**r**)] for (*a*) HCB, (*b*) PCP, (*c*) HBB and (*d*) PBP. *L*(**r**) plots are calculated in the planes defined by the positions of the three atoms involved in each synthon. CC and CD sites correspond to (3,−3) CPs (yellow) and (3,+1) CPs (pink) for all Cl and Br atoms. Intermolecular Cl⋯Cl and Br⋯Br contacts are shown with their bond paths (dotted lines) and the corresponding BCPs (small green circles) between the interacting atoms.

All the reported *X*⋯*X* interactions in C_6_Cl_6_, C_6_Cl_5_OH, C_6_Br_6_ and C_6_Br_5_OH (see Fig. 11 ▸) were found to be of ‘pure’ closed-shell type, since they showed |*V*|/*G* < 1 at their corresponding BCPs (Brezgunova *et al.*, 2012 ▸). The local properties of ρ(**r**) at BCPs indicate that the Cl⋯Cl and Br⋯Br interactions are weak, as shown by the small magnitudes of ρ and ∇^2^ρ (0.028 < ρ < 0.058 e Å^−3^ and 0.38 < ∇^2^ρ < 0.62 e Å^−5^ for Cl⋯Cl contacts, and 0.042 < ρ < 0.066 e Å^−3^ and 0.41 < ∇^2^ρ < 0.66 e Å^−5^ for Br⋯Br contacts, including both experimental and theoretical data). According to the local electronic properties at the BCPs, it was found that Br(δ^+^)⋯(δ^−^)Br interactions are more intense than Cl(δ^+^)⋯(δ^−^)Cl, owing to the larger electrophilic δ^+^ character of the heavier Br atom, even if the nucleophilic δ^−^ power of the lone pairs is smaller in the Br atom that for the lighter Cl atom.

**Figure 12:**
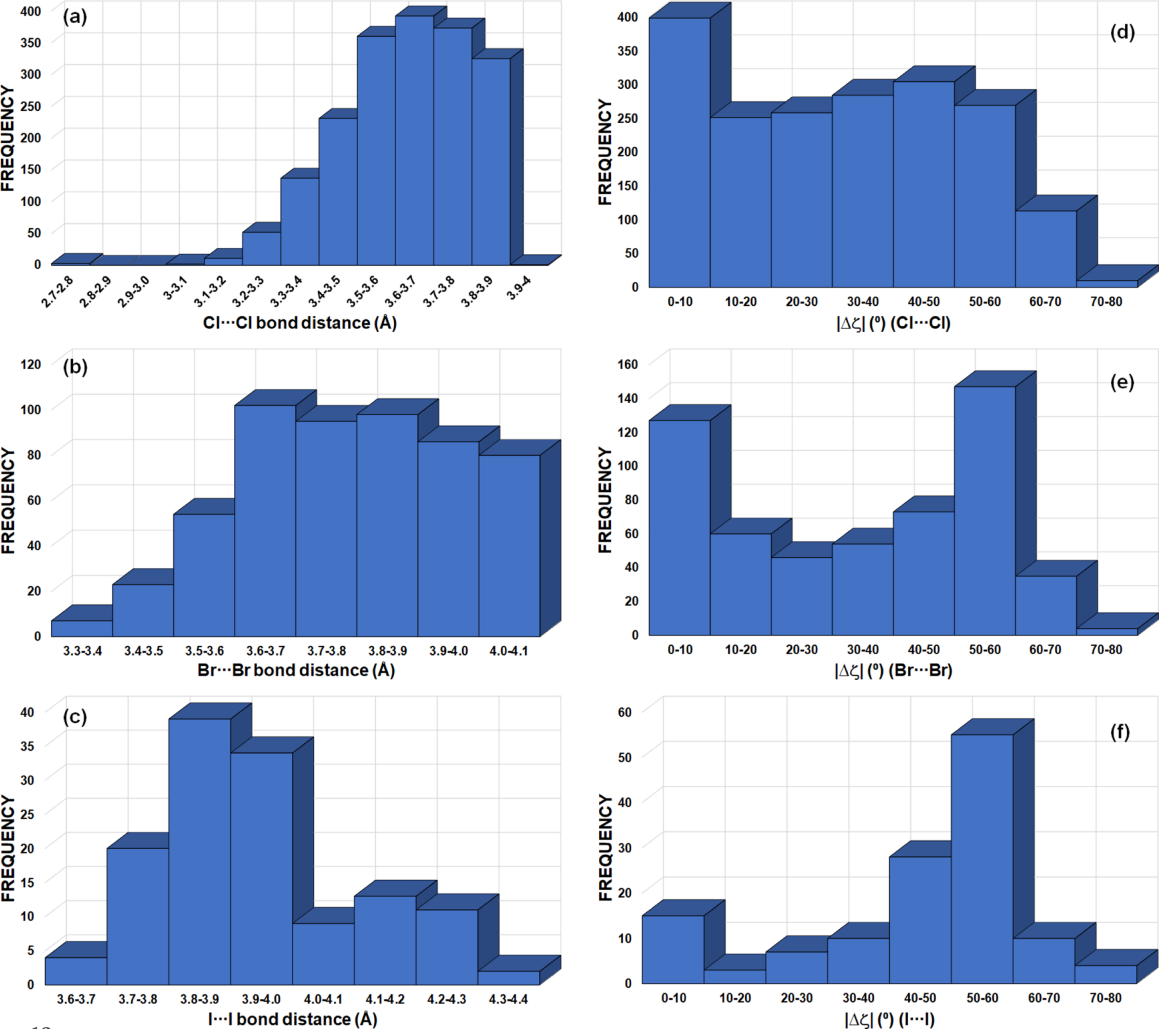
Frequency distributions for *X*⋯*X* (*X* = Cl/Br/I) XB interactions within homo-*X*_3_ synthons. For *X* = Cl, Br and I, the *X*⋯*X* distance and the |Δζ| angular orientation are represented in (*a*) and (*d*), (*b*) and (*e*), and (*c*) and (*f*), respectively. The motif shown in Fig. 10 ▸(*b*) was used for the CSD search with the geometrical parameters *d*(*X*⋯*X*) ≤ sum(vdW) + 0.4 Å and ζ_1_ and ζ_2_ angles within the range 100–180°. vdW radii = 1.75, 1.85 and 1.98 Å for Cl, Br and I atoms, respectively.

The CSD search for homo-*X*_3_ synthons (*X* = Cl/Br/I) resulted in 855 motifs and therefore in a total of 2565 *X*⋯*X* interactions (see the caption for Fig. 10 ▸ for the geometrical criteria). In Fig. 12 ▸, the peak in frequency observed for |Δζ| in the 0–10° range mainly corresponds to type-I halogen⋯halogen contacts, regardless of the type of halogen atom involved [Figs. 12 ▸(*d*)–12 ▸(*f*)]. In addition, the frequency plot for |Δζ| shows an extended maximum around 40–50° for *X* = Cl (1887 Cl⋯Cl interactions) and a clearly defined maximum at 50–60° for *X* = Br and I (546 Br⋯Br and 132 I⋯I interactions), as expected for XB interactions taking place in *X*_3_ synthons (Fig. 10 ▸). On the other hand, the frequency distribution of the *X*⋯*X* distances shows a peak for each type of halogen atom, appearing at 3.5–3.8, 3.6–3.9 and 3.8–4.0 Å for *X* = Cl, Br and I, respectively [Figs. 12 ▸(*a*)–12 ▸(*c*)]. These correspond approximately to the ranges 1 < *RR*_Cl_ < 1.3, 0.9 < *RR*_Br_ < 1.2 and 0.8 < *RR*_I_ < 1 (vdW radii = 1.75, 1.85 and 1.98 Å for *X* = Cl, Br and I, respectively), following the expected increase in interaction strength along the series Cl⋯Cl < Br⋯Br < I⋯I, which parallels the increasing polarizability Cl < Br < I of the halogen atoms.

The characterization of the topological CPs of the function *L*(**r**) shows that the CD⋯CC interactions are almost collinear with internuclear directions. Indeed, for the 12 *X*⋯*X* interactions found in the *X*_3_ synthons (*X* = Cl, Br) of the four crystal structures, the ranges for the angle between the two directions α(CD_1_⋯CC_2_; at_1_⋯at_2_) are 7.3–7.6, 10.5–12.2, 5.5–13.5 and 9.0–11.7° for HCB, HBB, PCP and PBP, respectively. This result further supports the fact that the relative orientation of atoms involved in intermolecular interactions, and therefore the supramolecular motifs they form, are driven by the local electrophilic⋯nucleophilic interactions between CD and CC sites, as previously observed in IDT, SePA and other crystal structures involving ChB interactions (Shukla *et al.*, 2020 ▸).

**Figure 13:**
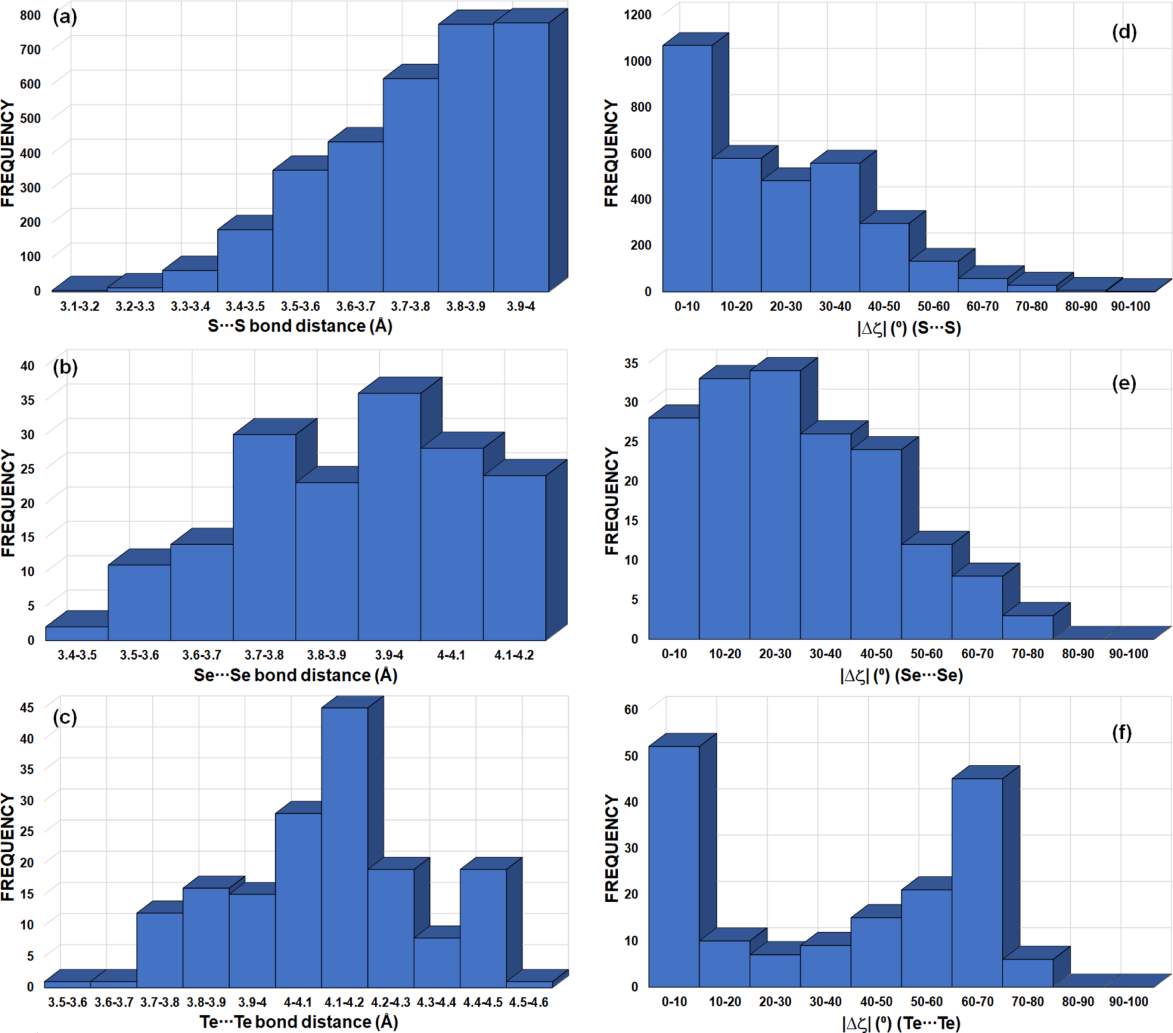
Frequency distributions in Ch⋯Ch (Ch = S/Se/Te) ChB interactions within homo-Ch_3_ synthons. For Ch = S, Se and Te, the Ch⋯Ch distance and the |Δζ| angular orientation are represented in (*a*) and (*d*), (*b*) and (*e*), and (*c*) and (*f*), respectively. The motif shown in Fig. 10 ▸(*c*) was used for the CSD search with the geometrical parameters *d* (Ch⋯Ch) ≤ sum(vdW) + 0.4 Å and ζ_1_ and ζ_2_ angles within the range 60–180°. vdW radii = 1.80, 1.90 and 2.06 Å for S, Se and Te atoms, respectively.

Triangular 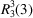 motifs involving chalcogen atoms are also found in the literature. For instance, they appear in some of the crystal structures of the families of 3*H*-1,2-benzodi­thiole-3-one and 3*H*-1,2-benzodi­thiole-3-thione heterocycles, and their selenium analogs (Shukla *et al.*, 2020 ▸). In order to expand the investigation of *X*_3_ synthons (*X* = Cl, Br, I) [Fig. 10 ▸(*b*)] to Ch_3_ synthons [Fig. 10 ▸(*c*)], we also performed a detailed CSD search for 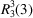 motifs involving Ch = S, Se and Te atoms in *sp*^3^ hybridization (Fig. 13 ▸). The search resulted in 1071, 56 and 55 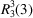 motifs with Ch = S, Se and Te atoms, leading to 3213 S⋯S, 168 Se⋯Se and 165 Te⋯Te interactions, respectively. The maxima in the frequencies for S⋯S, Se⋯Se and Te⋯Te distances [Figs. 13 ▸(*a*)–13 ▸(*c*)] are seen at 3.8–4.0, 3.9–4.0 and 4.1–4.2 Å, corresponding to 1.06 < *RR*_S_ < 1.11, 1.03 < *RR*_Se_ < 1.05 and 1.00 < *RR*_Te_ < 1.02 (vdW radii = 1.80, 1.90 and 2.06 Å for Ch = S, Se and Te, respectively). The peak in frequency observed for |Δζ| in the 0–10° range (for Ch = S/Te) corresponds mainly to type-I Ch⋯Ch contacts [Figs. 13 ▸(*d*) and 13 ▸(*f*)]. Besides this peak, the |Δζ| frequency plots show an extended maximum at 20–30° for Ch = Se, a small maximum at 30–40° for Ch = S and a very well defined maximum at 60–70° for Ch = Te. The *RR* results are in line with those of *X*_3_ synthons, whereas the frequency plots of |Δζ| do not show peaks at high |Δζ| angles for all Ch atoms that could be systematically associated to electrophilic⋯nucleophilic interactions, as in *X*_3_ synthons. However, as discussed in Section 4.2.3 and due to the presence of multiple δ^+^/δ^−^ sites in Ch_*sp*^3^_ atoms [Fig. 4 ▸(*a*)], the structural descriptors associated with Ch^δ^^+^⋯^δ^^−^Ch ChB interactions need to be handled with care. According to our previous analysis of Ch⋯Ch contacts, |Δζ| ≥ 50° mainly encompasses electrophilic⋯nucleophilic (δ^+^⋯δ^−^) ChB interactions, even if some type-I and stacking interactions can still be found in this particular region [Figs. 8 ▸(*a*) and 9 ▸(*a*)]. Hence, due to the very well defined frequency maxima occurring at |Δζ| = 60–70°, Te is found to be the most suitable amongst the chalcogens for forming Ch_3_ synthons.

On the other hand, besides *X*_3_ synthons and Ch_3_ synthons, other triangular 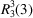 motifs based on pnictogen-bonded cyclic trimers (PH_2_*Y*)_3_ (*Y* = F, Cl, OH, CN, NC, CH_3_, H and BH_2_) with *C*_3h_ symmetry and bonding energies ranging from −17 to 63 kJ mol^−1^ have also been described in the literature from theoretical calculations (Alkorta *et al.*, 2013 ▸). However, they are beyond the scope of this study, which mainly focuses on ChB, XB and HB interactions, and will not be addressed here.

#### 4.3.2. Bifurcated V-type motifs

The most common V-type atomic motifs are found in HB interactions H⋯*Y* (*Y* = O, N), bifurcated at either *Y* or H. They involve local δ^+^⋯ δ^−^ interactions, where δ^+^ corresponds to a CD region along the extended bonding direction of H and δ^−^ corresponds to a lone-pair region of *Y*. Accordingly, several very common situations can appear: (i) the δ^+^ region of a single H atom interacts with δ^−^ regions of two *Y* atoms, (ii) a single δ^−^ region of one *Y* atom interacts with each δ^+^ region of two H atoms, and (iii) two δ^−^ regions of a single *Y* atom interact with δ^+^ regions of two H atoms. Hereafter we will see that V-type motifs can also be found with other type of atoms, as long as they contribute with appropriate δ^+^ or δ^−^ regions and have a convenient angular disposition in the δ^+^⋯δ^−^ interactions building the motif.

In the crystal structure of IDT, a bifurcated V-type motif involving HB (H⋯O) and XB (I⋯O) interactions has been identified [Figs. 2 ▸(*a*) and 14 ▸(*a*)]. Table 4 ▸ presents the structural parameter characteristics of this motif. Here, the O atom forms a bifurcated interaction with H and I atoms, showing almost equal ζ_2_ angles (∠C—O⋯H/I = 137.8/137.5°) that are larger than those typically observed for the location of the oxygen lone pairs in *sp*^2^ hybridization (∼120°). This can be explained by the need to respond to the arrangement of two neighbouring molecules involved in the intermolecular interactions. On the other hand, as expected, the angle ζ_1_ for C—I⋯O is closer to linear geometry (170.3°) than for C—H⋯O (158.6°). With respect to the *RR* parameter, the two interactions present very similar values (0.82 and 0.84).

**Figure 14:**
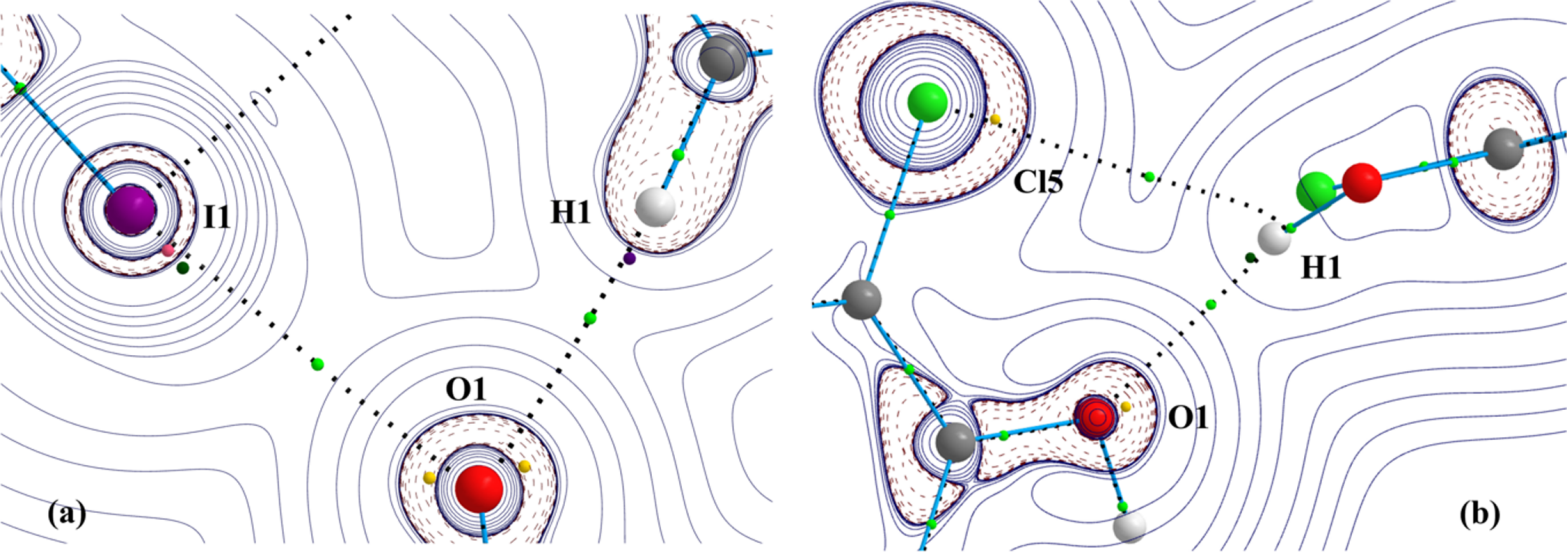
Maps and topological CPs of the function *L*(**r**) [= −∇^2^ρ(**r**)] for (*a*) IDT and (*b*) PCP. CC sites correspond to (3,−3) CPs (yellow) for O and Cl atoms, CD sites correspond to either (3,+3) (violet) CPs for H atoms, or (3,−1) (dark green) CPs for H and I atoms. Intermolecular HB and XB interactions (*X*^δ^^+^⋯^δ^^−^O, with *X* = H, I) are shown with their bond paths (dotted lines) and the corresponding BCPs (small light-green circles) between the interacting atoms. Planes are defined by the interacting atoms in (*a*) and by the CPs of *L*(**r**) focused on in (*b*).

Fig. 14 ▸(*b*) shows another bifurcated V-type motif involving O—H⋯O—C and O—H⋯Cl—C interactions, previously observed in the reported crystal structure of penta­chloro­phenol (C_6_Cl_5_OH, PCP) (Brezgunova *et al.*, 2012 ▸). The O—H⋯O—C interaction is a short hydrogen bond (*RR* = 0.74), with structural angles of ζ_1_ = 151.3° and ζ_2_ = 125.4° indicating the expected electrophilic and nucleophilic regions close to the O—H bonding direction and around the oxygen lone pair in *sp*^2^ hybridization. The O—H⋯Cl—C interaction exhibits a distance close to a vdW contact (*RR* = 0.99), with structural angles of ζ_1_ = 114.7° and ζ_2_ = 89.3°, suggesting that the δ^+^ region of the H atom is involved in a lateral interaction with the δ^−^ region of the Cl atom lone pair that is perpendicular to the C—Cl bonding direction (Table 4 ▸).

The bond paths and BCPs of the V-type motifs found in the trimer of IDT and in the dimer of PCP are depicted in Fig. 14 ▸, along with the relevant CC and CD sites found from the topological analysis of the function *L*(**r**). Structural data, electron properties at BCPs, estimated interaction energies and the angular descriptor α between the CC⋯CD and internuclear directions are presented in Table 4 ▸.

In both bifurcated V-motifs the interactions are of ‘pure’ closed-shell type (|*V*|/*G* < 1) (Table 4 ▸) (Espinosa *et al.*, 2002 ▸). In IDT, the topological (ρ, ∇^2^ρ) and local energetic properties (*G*, *V*, |*V*|/*G*) at BCPs are larger for O⋯I than for O⋯H, indicating a stronger interaction in the former. In addition, from the estimated energetic contributions to the dimer interaction energy (*E*_int_), the O⋯I bonding is more energetic than O⋯H. On the other hand, in PCP, the magnitudes of ρ, ∇^2^ρ, *G*, *V* and |*V*|/*G* are significantly larger for H⋯O than for H⋯Cl (Table 4 ▸), in particular when looking at their estimated energy contributions to the interaction energy of the dimer (*E*_int_). As in the previous sections dealing with 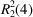 and 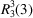 motifs, the characterization of the topological CPs of the function *L*(**r**) in the V-type motifs shows local electrophilic⋯nucleophilic interactions (CD⋯CC) that are remarkably well aligned with internuclear directions. Indeed, α < 10° for the four interactions in the V-type motifs. A second value of α is also given for the H⋯O interaction in PCP to emphasize that the π plane containing both lone pairs is in fact directed towards H (the second α value corresponds to the second CC site of O). It is also noticeable that, despite the very weak H⋯Cl interaction (pointed out by all the topological properties at the BCP), the angle α is lower than 10°. This result seems to indicate that directionality is not necessarily linked to a significant interaction energy. A similar finding is observed, for instance, in *X*_3_ synthons. In the I⋯O interaction of IDT, a (3,+1) CP of *L*(**r**) is also plotted in Fig. 14 ▸(*a*), which is found further inside the electron shell than the (3,−1) CP associated with the CD site of I. The two CPs are connected by a gradient line of *L*(**r**) that indicates the direction of electrophilicity of the atom, while the angle α is very similar for both of them. Their joint representation aims to show that, in cases of heavier atoms such as I, the CD site is better described by a (3,−1) CP of *L*(**r**) in a region of large depletion of electron density rather than the usual (3,+1) CP, which is found in a region of significant electron concentration [∇^2^ρ(**r**) < 0].

#### 4.3.3. Intramolecular interactions

**Figure 15:**
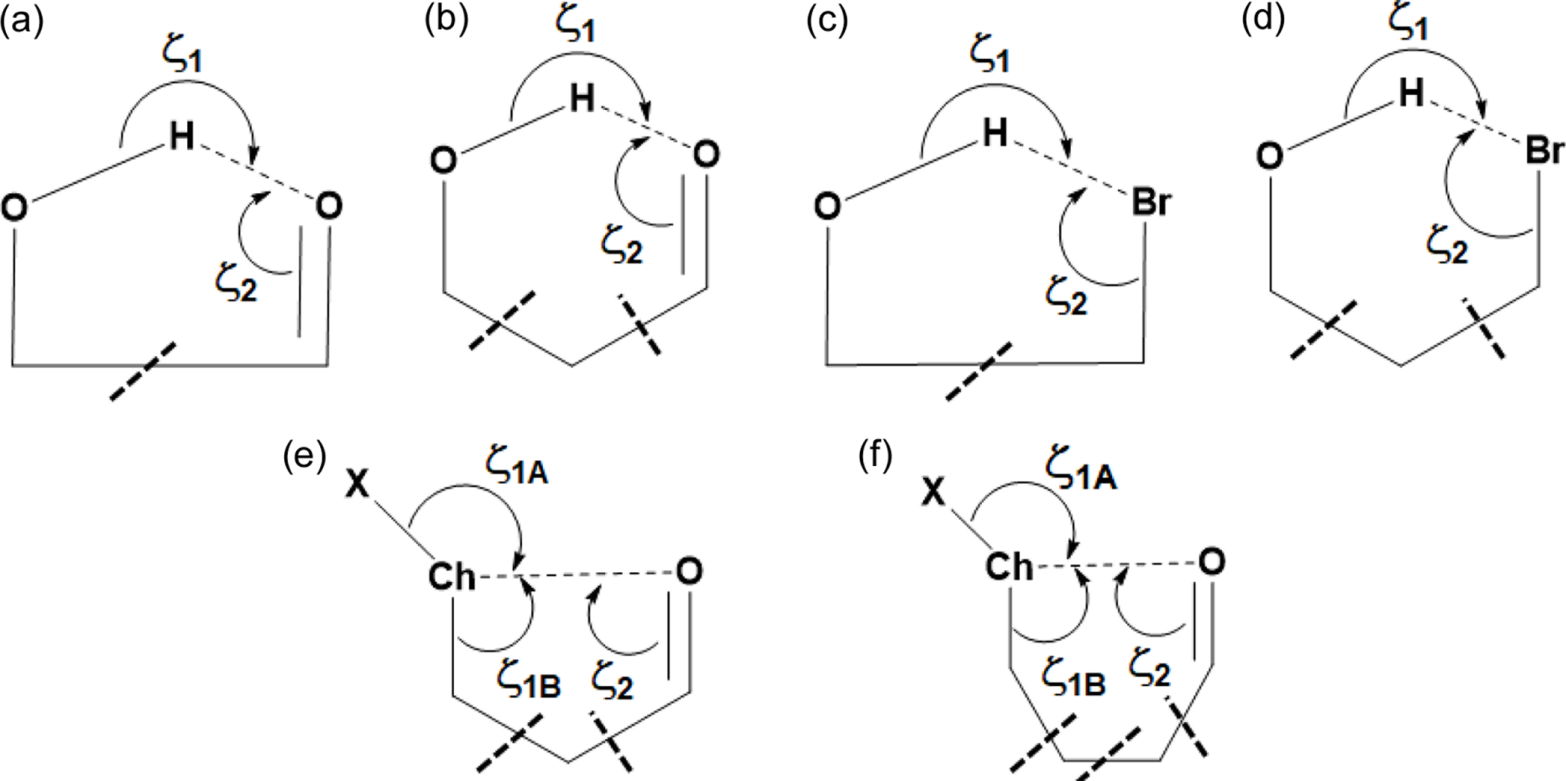
CSD searches performed for non-covalent intramolecular interactions involving (*a*) H⋯O hydrogen bonds belonging to 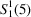 motifs, (*b*) H⋯O hydrogen bonds belonging to 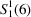 motifs, (*c*) H⋯Br hydrogen bonds belonging to 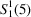 motifs, (*d*) H⋯Br hydrogen bonds belonging to 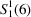 motifs, (*e*) Ch⋯O chalcogen bonds (Ch = S, Se) belonging to 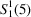 motifs with *X* = any atom, and (*f*) Ch⋯O chalcogen bonds (Ch = S, Se) belonging to 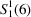 motifs with *X* = any atom. Dashed lines crossing the bonds represent bonds of any type (single, double, aromatic *etc.*). Geometric criteria used for the CSD search were: distances H⋯O/H⋯Br/Ch⋯O ≤ sum(vdW radii), angles ζ_1_ = ∠O—H⋯O/∠O—H⋯Br, ζ_2_ = ∠C=O⋯H/∠C=O⋯Br/∠C=O⋯Ch, ζ_1A_ = ∠*X*—Ch⋯O and ζ_1B_ = ∠C—Ch⋯O ranging from 60 to 180° (vdW radii = 1.20, 1.52, 1.80, 1.90 and 1.85 Å for H, O, S, Se and Br atoms, respectively).

The results of CSD searches for intramolecular HBs and ChB interactions are shown in Fig. 15 ▸. Differing from the former notation of Etter (1990 ▸), where the graph-set notation *S*(*n*) denotes an intramolecular HB pattern of *n* members and subscript *d* and superscript *a* do not appear because they equal 1 in all cases, in the new graph-set notation involving CC and CD sites, it is of interest to keep both the subscript *e* and superscript *n* to describe the number of electrophilic and nucleophilic sites in the interaction, as shown later.

The most common non-covalent intramolecular interactions concern H⋯O hydrogen bonds, forming 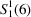 motifs. 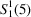 motifs involving H⋯O hydrogen bonds are also observed, but are in less number. Supporting this observation, a CSD search for these motifs gave rise to 3596 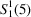 and 9793 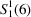 hits. On the other hand, a similar search involving H⋯Br hydrogen bonds resulted in 295 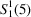 and 10 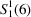 hits. Based on the total number of hits, these results indicate that O is more commonly observed as a hydrogen-bond acceptor than Br for either the 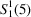 or the 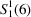 motif. In the case of Br, the larger number of hits involving 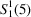 motifs [with respect to 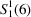 motifs] may indicate that larger atoms accommodate better with the geometry of five-membered rings than smaller atoms, such as O, for which the intramolecular non-covalent interaction appears to be favoured within a six-membered ring geometry, as shown by the number of hits for 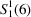 and 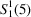 motifs involving H⋯O contacts. A similar feature is observed in intramolecular chalcogen bonding, where larger donor atoms (such as Ch = S, Se with *r*_vdW_ = 1.80, 1.90 Å, respectively) favour the obervation of a greater number of five-membered rings with respect to six-membered rings. Indeed, the search for intramolecular Ch⋯O chalcogen bonds resulted in 1501 and 183 hits for 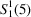 and 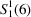 motifs, respectively, with Ch = S, whereas the search resulted in 152 and 5 hits for 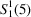 and 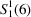 motifs with Ch = Se. Hence, with S, Se and Br atoms, the number of 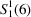 motifs are only 12, 3 and 3% of the number of observed 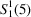 motifs with the same type of atoms, respectively, whereas the opposite is observed for O atoms where the number of 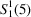 motifs are 37% of the number of 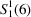 motifs.

**Figure 16:**
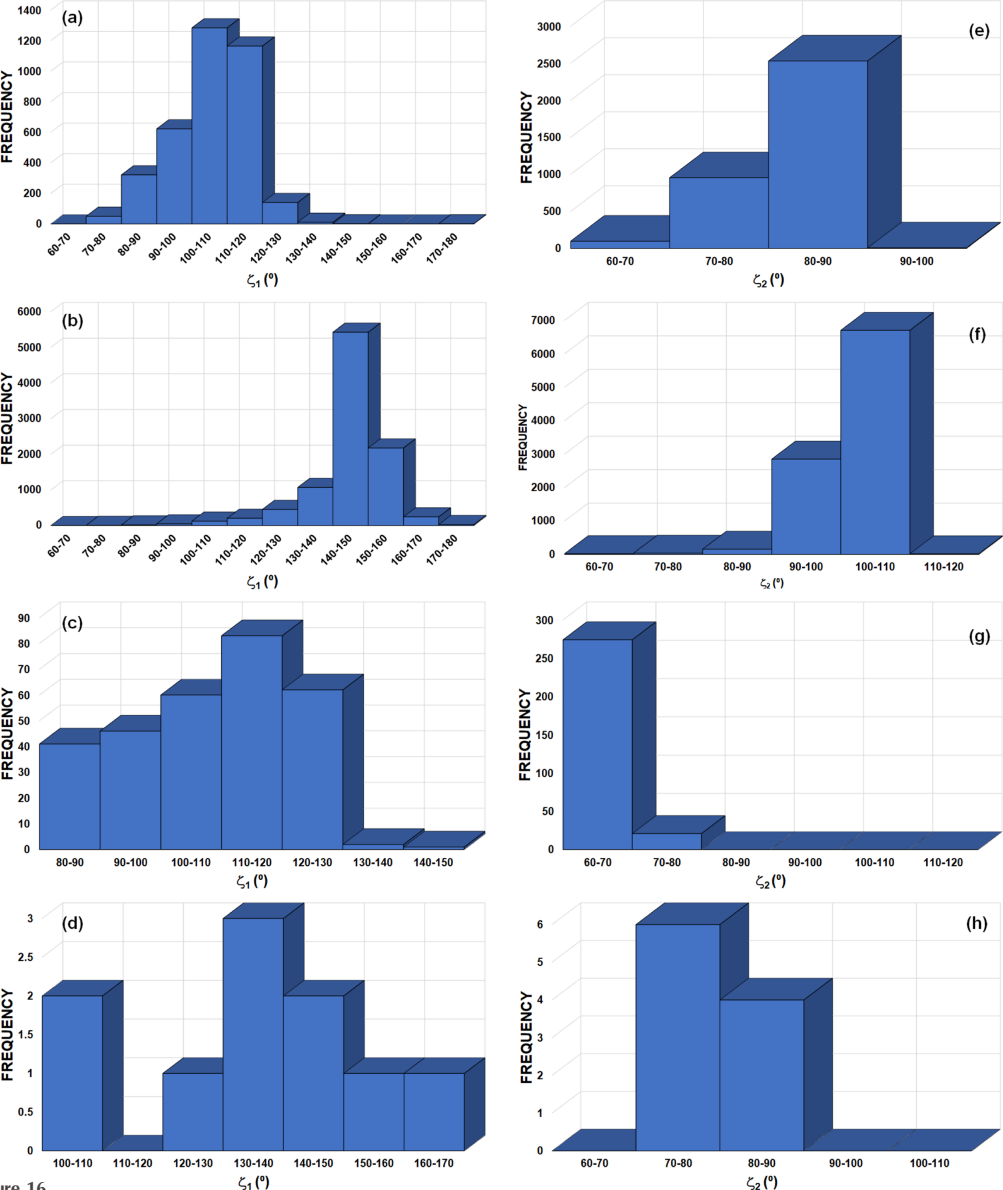
ζ_1_ frequency distribution for HB interactions: (*a*) 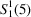 H⋯O, (*b*) 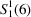 H⋯O, (*c*) 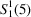 H⋯Br and (*d*) 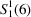 H⋯Br. ζ_2_ frequency distribution for HB interactions: (*e*) 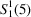 H⋯O, (*f*) 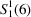 H⋯O, (*g*) 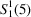 H⋯Br and (*h*) 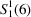 H⋯Br.

**Figure 17:**
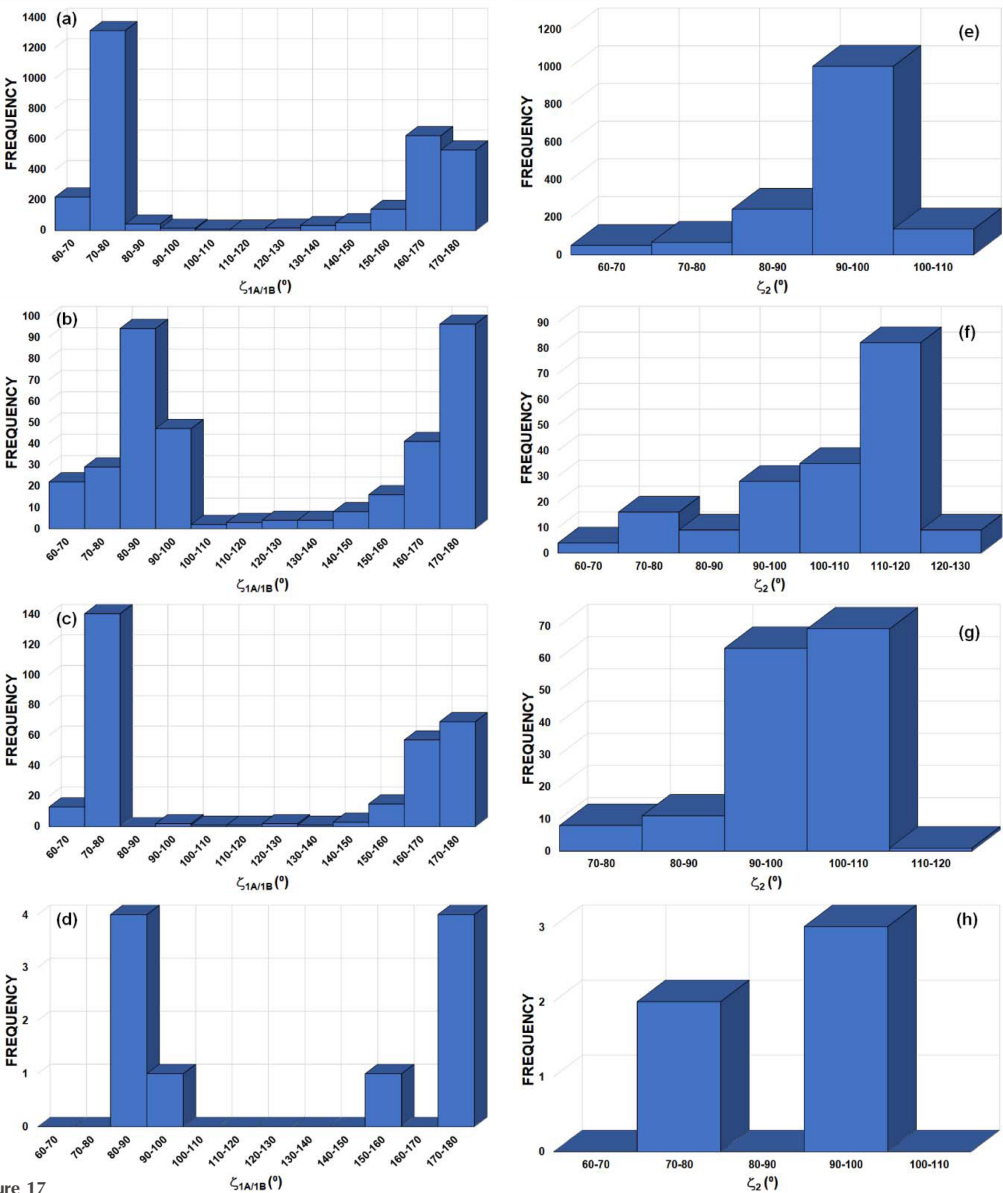
ζ_1A/1B_ frequency distribution for ChB interactions: (*a*) 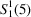 S⋯O, (*b*) 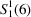 S⋯O, (*c*) 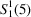 Se⋯O and (*d*) 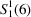 Se⋯O interactions. ζ_2_ frequency distribution for ChB interactions: (*e*) 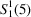 S⋯O, (*f*) 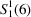 S⋯O, (*g*) 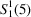 Se⋯O and (*h*) 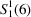 Se⋯O.

Fig. 16 ▸ shows the frequency distribution of the ζ_1_ and ζ_2_ angles defined in Fig. 15 ▸ for H⋯O and H⋯Br HB interactions forming 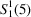 and 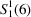 motifs. For both types of hydrogen bonds, the frequency peaks shift towards larger angles from 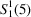 (ζ_1_ = 100–110° and ζ_2_ = 80–90° for H⋯O, and 110–120° and ζ_2_ = 60–70° for H⋯Br) to 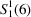 motifs (ζ_1_ = 140–150° and ζ_2_ = 100–110° for H⋯O, and ζ_1_ = 130–140° and ζ_2_ = 70–80° for H⋯Br). A similar result is observed for the corresponding frequency distribution of the ζ_1A/1B_ and ζ_2_ angles defined in Fig. 15 ▸ for S⋯O and Se⋯O ChB interactions (Fig. 17 ▸). Indeed, they shift to larger angles from 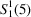 (for S⋯O: ζ_1A_ = 160–170°, ζ_1B_ = 70–80° and ζ_2_ = 90–100°; for Se⋯O: ζ_1A_ = 160–180°, ζ_1B_ = 70–80° and ζ_2_ = 90–110°) to 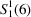 motifs (for S⋯O: ζ_1A_ = 170–180°, ζ_1B_ = 80–90° and ζ_2_ = 110–120°; for Se⋯O: ζ_1A_ = 170–180°, ζ_1B_ = 80–90° and ζ_2_ = 90–100°), even if the very few cases of 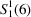 motifs with Se⋯O interactions cannot be considered statistically significant. While the ζ_1A_ angle of ∼180° in both S⋯O and Se⋯O interactions mostly seems to indicate that the electrophilic region along the C—Ch bond [in both 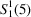 and 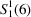 motifs] is responsible for the chalcogen bond, the electrophilic region along the O—H bonding direction is clearly better oriented in 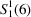 motifs (ζ_1_ = 140–150°) than in 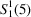 motifs (ζ_1_ = 100–110°), in line with the previous observation that showed a larger number of hits with the former motif. Additionally, in both 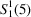 and 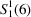 motifs involving either HB or ChB interactions, the ζ_2_ angle approximately corresponds to the expected position of a lone pair of the acceptor atom (O or Br) in its particular hybridization.

**Figure 18:**
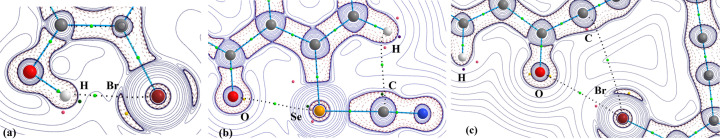
Maps and topological CPs of the function *L*(**r**) [= −∇^2^ρ(**r**)] for (*a*) H⋯Br in PBP, (*b*) Se⋯O and H⋯C in AHEQIN and (*c*) Br⋯O and C⋯Br in GOHJAQ. CC sites correspond to (3,−3) CPs (yellow) for O and Br atoms and to (3,−1) CPs (dark green) for the C atom; CD sites correspond to (3,+1) (pink) and (3,−1) CPs (dark green) for H atoms, (3,+1) CPs (pink) for the C atom, (3,−1) CPs (dark green) for the Se atom and (3,+1) CPs (pink) for the Br atom. Intramolecular HB, ChB, XB and TrB interactions [*X*(δ^+^)⋯(δ^−^)*Y* with *X* = H, Se, Br; *Y* = O, Br] are shown with their bond paths (dotted lines) and their corresponding BCPs (small light-green circles) between the interacting atoms.

The crystal structure of PBP (Brezgunova *et al.*, 2012 ▸) exhibits an intramolecular H⋯Br HB interaction [Fig. 18 ▸(*a*)]. As presented in Table 5 ▸, the interaction shows a significantly low reduction ratio parameter *RR* = 0.75, while the associated structural angles ζ_1_ and ζ_2_ (146.1 and 62.9°) indicate an HB interaction with an O—H^δ^^+^ electrophilic region well oriented towards the nucleophilic region of the Br lone pair, forming an 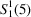 motif [Figs. 16 ▸(*c*) and 16 ▸(*g*)]. In line with the large ζ_1_ angle, the short HB distance and the small *RR* value, the H⋯Br interaction exhibits an incipient degree of covalence |*V*|/*G* >1 with a significant electron density magnitude at the BCP (ρ = 0.232 e Å^−3^).

For comparison with other intramolecular HB patterns, we have also performed topological analysis on intramolecular chalcogen and hydrogen bonding [Fig. 18 ▸(*b*)], and halogen and tetrel bonding [Fig. 18 ▸(*c*)]. Thus, two 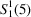 motifs sharing a C—Se moiety (CSD code AHEQIN; Jones *et al.*, 2002 ▸) and a pair of 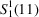 and 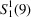 motifs intersecting on a secondary 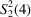 motif (GOHJAQ, Widner *et al.*, 2014 ▸) were studied. Note that all of them are found in the molecular planes. The scheme below[Chem scheme2] shows the expected positions of the electrophilic and nucleophilic regions building the two 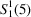 motifs in AHEQIN, and the 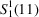 and 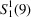 motifs [intersecting on a secondary 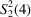 motif] in GOHJAQ, as discussed below. From the structural parameters (Table 5 ▸), the significantly low *RR* values demonstrate that these intramolecular interactions are very short (except for the tetrel bond, TrB), while the ζ_1_ and ζ_2_ angles further confirm the expected geometry of the electrophilic⋯nucleophilic interaction involved in the intramolecular chalcogen and halogen bonding under investigation. In Fig. 18 ▸(*c*), the V-type bifurcated interaction at the amphoteric halogen atom involves its electrophilic σ-hole region along the C—Br bonding direction in a halogen bond (C—Br^δ^^+^⋯^δ^^−^O) and its nucleophilic lone-pair region in the tetrel bond (C—C^δ^^+^⋯^δ^^−^Br), leading to an 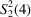 motif. The existence of these non-covalent intramolecular interactions is established by the presence of bond paths and the concomitant BCPs (Fig. 18 ▸). In AHEQIN and GOHJAQ, the three intramolecular XB, HB and TrB interactions are of ‘pure’ closed-shell type (|*V*|/*G* < 1), whereas the ChB interaction shows an incipient degree of covalence (|*V*|/*G* > 1). The topological parameters at the BCP of the Se⋯O chalcogen bond in AHEQIN are roughly similar to those calculated for the H⋯Br hydrogen bond in PBP, suggesting similar strength. In comparison, the intramolecular XB (Br⋯O) and TrB (C⋯Br) interactions in GOHJAQ are observed to be significantly weaker (Table 5 ▸).[Chem scheme2]
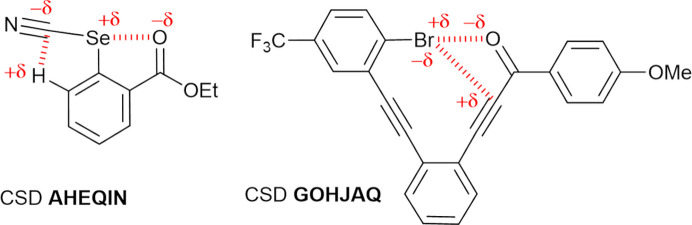


As previously pointed out, the assignment of CC and CD sites to specific CPs of the function *L*(**r**) should be taken with care. Hence, in addition to the CPs that are associated with CC and CD sites, surrounding CPs are plotted in Fig. 18 ▸ for discussion. Nucleophilic sites are clearly identified by the (3,−3) CPs that are associated with the atomic lone pairs of Br and O atoms, whereas in π systems they appear as (3,−1) CPs, such as in the H⋯C interaction of AHEQIN. On the other hand, π systems also develop electrophilic sites that are described by (3,+1) CPs, such as in the C⋯Br interaction of GOHJAQ. Because of the development of (3,−1) and (3,+1) CPs in π systems, the local electrostatic complementarity of electrophilic⋯nucleophilic interactions also takes place in π–π interactions and drives the mutual arrangement of these systems. This trend, being beyond the scope of this work, has not been investigated in depth here and will be addressed in future research. The electrophilic site of an H atom is typically described by a (3,+3) CP that appears along the *X*—H bond direction. Concomitantly, it is very common to find two (3,+1) CPs, one on each side of this (3,+3) CP, such as in AHEQIN. These (3,+1) CPs are typically involved in lateral HB interactions, as observed in the H⋯C interaction of this compound and in the orientation of the (3,+1)⋯(3,−3) CPs with the internuclear direction H⋯O of GOHJAQ. In the case of PBP, and due to the strong H⋯Br interaction that pulls the O—H group toward the intramolecular region, the electron distribution around the H atom follows the effect of the surrounding atoms, leading to the coalescence of the (3,+3) and (3,+1) CPs, which disappear while the observed (3,−1) CP emerges in the interaction with the (3,−3) CP of the Br atom. The σ-hole interaction of Se with the lone pair of O in AHEQIN involves a (3,−1) CP that behaves as a CD site because the lone pairs of the Se atom are sufficently separated, as explained in previous sections. This feature permits the development of an extended σ plane of electrophilic character in front of the O atom where, in addition to the (3,−1) CP, we also found two (3,+1) CPs, one on each side of the former, as observed in SePA and other chalcogenated molecules (Shukla *et al.*, 2020 ▸). In GOHJAQ, the σ hole of Br shows an electrophilic region more directed toward the lone pair of the O atom. The corresponding CD site is assigned to a (3,+1) CP because the (3,−1) CP found for the heavier I atom in IDT does not appear here for Br.

The topological CPs of the function *L*(**r**) in the intramolecular motifs analysed again show that CD⋯CC interactions are almost collinear with internuclear directions (α ≤ 15° for all of them, see Table 5 ▸). Note that, in addition to the observations made for intermolecular motifs and synthons, they also indicate that local electrophilic⋯nucleophilic interactions between CD and CC sites facing each other drive the atomic orientation in intramolecular motifs.

## 5. Conclusions

It is quite difficult to predict the geometrical preferences of intermolecular interactions involving chalcogen atoms because the position of their electrophilic (δ^+^) and nucleophilic (δ^–^) regions can appear enlarged and/or shifted depending on the chalcogen atom type and on its atomic environment. Indeed, in addition to a σ-hole region that can be found enlarged in the σ plane (encompassing a wider range of contact angles than, for instance, in XB interactions, and therefore blurring the expected directionality of the ChB interactions along bonding directions), the relative position of its lone pairs in the π plane can lead to either a δ^+^ or a δ^–^ region close to the intersection of the π and σ planes. The structural angles are very useful for characterizing the geometrical particulars of their intermolecular contacts and therefore for analysing electronic interactions involving chalcogen atoms in *sp*^3^ hybridization. To this end, the geometrical descriptors Δζ = ζ_1_ − ζ_2_ and Δϕ = ϕ_1_ − ϕ_2_ defined in this work and based on (i) the intermolecular contact angles ζ_i_ = max(ζ_iA_, ζ_iB_), (ii) the intramolecular angles α_*i*_ = C_*i*A_—Ch_*i*_—C_*i*B_ and (iii) the planarity degree angle in the σ_*i*_ plane ϕ_*i*_ = ζ_*i*A_ + ζ_*i*B_ + α_*i*_ (*i* = 1, 2), have shown their usefulness. With this respect, the Δϕ versus Δζ plot is a very convenient way to differentiate between different Ch⋯Ch contacts, and in particular to assess δ^+^⋯ δ^–^ ChB interactions.

The CSD searches and frequency plots of geometrical descriptors of synthons and other supramolecular motifs highlight the particular orientations of intermolecular interactions in all the supramolecular structures investigated in this work. These specific orientations match the expected positions of electrophilic (δ^+^) and nucleophilic (δ^–^) regions in either ChB, XB or HB interactions.

Molecular δ^+^ and δ^−^ regions are observed in the deformation Δρ(**r**) maps in the external part of the molecules. The electronic sites responsible for these features are found in the valence shells of the atoms and are assigned by the critical points of the *L*(**r**) function, which determine their either CD or CC character along their three main topological directions. The topological analyses of ρ(**r**) and *L*(**r**) functions point out that the observation of particular supramolecular motifs does not depend on the type of atom and functional group involved in donor and acceptor sites, and therefore on the bonding type (such as, for instance, ChB, XB, HB or TrB). Indeed, once relevant electrophilic and nucleophilic regions are placed facing each other along the internuclear directions of the intermolecular interactions that assemble the structural motif, the motif forms in a similar way. As an example, the supramolecular four-membered motif observed in IDT and SePA is electronically equivalent in the two crystal structures, building a synthon that is independent of the atoms involved.

The proposed graph-set assignment 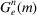 (*G* = *C*, *R*, *D* or *S*) is a very convenient way to describe the connectivity in motifs based on electrophilic⋯nucleophilic interactions, where *n* and *e* denote the number of nucleophilic (CC) and electrophilic (CD) sites, and *m* is the number of atoms building the motif. In all the 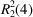, 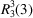, V-type, and 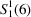, 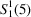 and 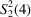 motifs explored, involving either inter- or intramolecular ChB, XB, HB or TrB interactions, the orientation of atoms and functional groups is systematically driven by local electrostatic electrophilic⋯nucleophilic interactions between CD⋯CC sites, as suggested by the small α angle between CD⋯CC and the internuclear directions (in most cases α < 15°). Hence, CC and CD sites drive the relative positions of molecules by orienting their atoms. They are thus at the origin of particular molecular assemblies, governing the geometrical preferences of synthons and other supramolecular motifs despite the fact that they can exhibit modest interaction energies. According to the features described in this work, the local complementary nature of CD⋯CC interactions makes them the building blocks of recurring supramolecular motifs, even if made from different atoms and functional groups and embedded in different molecular environments.

This work will be followed by further investigations dealing with descriptors measuring the nucleophilic/electrophilic power of CC/CD sites and the interaction between them. In addition to synthons and supramolecular motifs, we would also like to extend this analysis to π⋯π stacking interactions in the same framework. In our future work, a central point of interest will concern the sensitivity of the geometrical position of CC/CD sites, and their nucleophilic/electrophilic power, to molecular environments and conformations.

## Supplementary Material

Crystal structure: contains datablock(s) I. DOI: 10.1107/S2052252525001447/pen5012sup1.cif

Structure factors: contains datablock(s) I. DOI: 10.1107/S2052252525001447/pen5012sup2.hkl

Supporting tables and figure. DOI: 10.1107/S2052252525001447/pen5012sup3.pdf

CCDC reference: 2424586

## Figures and Tables

**Table 1:** Structural parameters (see Fig. 4 ▸) characterizing Ch(δ^+^)⋯(δ^−^)*Y* (Ch = S, Se; *Y* = O, S, I) chalcogen-bonding interactions in IDT and SePA Parameters with units: distance (*d*) Ch⋯*Y* (*Y* = Ch, *X*) (Å); *RR*, defined as the Ch⋯*Y* distance over the sum of vdW radii (dimensionless); contact angles C_1A_—Ch_1_⋯*Y*, ζ_1A_ (°), C_1B_—Ch_1_⋯*Y*, ζ_1B_ (°), Ch_1_⋯*Y*—C_2A_, ζ_2A_ (°) and Ch_1_⋯*Y*—C_2B_, ζ_2B_ (°); molecular angles C_1A_—Ch_1_—C_1B_, α_1_ (°) and C_2A_—Ch_2_—C_2B_, α_2_ (°); degree of planarity angle in the σ_*i*_ plane ϕ_*i*_ = ζ_*i*A_ + ζ_*i*B_ + α_*i*_, where *i* = 1, 2 (°); ζ_1_ = max(ζ_1A_, ζ_1B_) and ζ_2_ = max(ζ_2A_, ζ_2B_). vdW radii = 1.52, 1.80, 1.90 and 1.98 Å for O, S, Se and I, respectively.

Compound	Ch(δ^+^)⋯(δ^–^)*Y*†	*d*	*RR*	ζ_1A_/ζ_1B_	α_1_	ϕ_1_	ζ_2A_/ζ_2B_	α_2_	ϕ_2_	ζ_1_ − ζ_2_	ϕ_1_ − ϕ_2_
IDT	S_1_^i^⋯S_2_^iii^	3.8308 (1)	1.06	161.4/97.9	96.0	355.3	126.6/135.4	96.6	358.6	26.0	−3.3
	S_2_^iii^⋯I_1_^i^	3.8035 (3)	1.01	171.0/84.9	96.6	352.5	92.7	–	–	78.3	–
SePA	Se_1_^i^⋯Se_1_^ii^	3.8220 (2)	1.01	166.3/79.1	87.4	332.8	87.4/122.0	87.4	296.8	44.3	36
	Se_1_^i^⋯O_1_^ii^	3.3552 (1)	0.98	119.0/145.4	87.4	351.8	111.2	–	–	34.2	–

† Symmetry codes. IDT: (i) *x*, *y*, *z*; (iii) *x*, *y*, −1 + *z*. SePA: (i) *x*, *y*, *z*; (ii) 1/2 − *x*, −1/2 + *y*, 3/2 − *z*.

**Table 2:** Structural and topological parameters for Ch(δ^+^)⋯(δ^−^)*Y* (Ch = S, Se; *Y* = O, S, Se, I) chalcogen-bonding interactions in IDT and SePA α is the angle between the CD⋯CC and internuclear directions. Parameters with units: α (°), topological and local energetic properties ρ(e Å^−3^), ∇^2^ρ (e Å^−5^), *G* and *V* (kJ mol^−1^ bohr^−3^), and |*V*|/*G* (dimensionless), calculated at BCPs using the *AIMAll* software (Keith, 2019 ▸) with interacting dimers calculated at experimental geometries. vdW radii = 1.52, 1.80, 1.90 and 1.98 Å for the O, S, Se and I atom, respectively.

Compound	Ch(δ^+^)⋯(δ^−^)*Y*†	ρ	∇^2^ρ	*G*	*V*	|*V*|/*G*	−*E*_int_‡	α§
IDT	S_1_^i^⋯S_2_^iii^	0.028	0.32	6.6	−4.2	0.64	2.1/–	10.1
	S_2_^iii^⋯I_1_^i^	0.048	0.45	10.0	−7.6	0.76	3.8/–	8.7
	Dimer *E*_int_						6.4/5.9/5.9	
SePA	Se_1_^i^⋯Se_1_^ii^	0.041	0.36	8.1	−6.3	0.78	3.2/4.7	17.3/15.0
	Se_1_^i^⋯O_1_^ii^	0.050	0.60	12.9	−9.4	0.73	4.7/5.9	11.7
	Dimer *E*_int_						9.4/8.3/7.9/10.6	

† See Table 1 ▸ for symmetry codes.

‡ For individual interactions, −*E*_int_ (kJ mol^−1^) is estimated as (left) −*V*/2 and (right) −0.375*V* + 2.366 [for ChB interactions involving Se atoms, see Bauzá & Frontera (2020 ▸)]. The interaction energy of the dimers (difference between the energy of the dimer and those of the monomers) has been calculated at the B3LYP-D3/Def2TZVPP level of theory: values (in kJ mol^−1^) correspond to −*E*_int_dimer_/−*E*_int_dimer_corrected_BSSE_/−Σ*V*/2/−Σ*E*_int_(Bauzá & Frontera, 2020 ▸)_.

§ In the case of the Se_1_^i^⋯Se_1_^ii^ interaction, one CC seems to interact simultaneously with two CD sites, leading to two α angles.

**Table 3:** Halogen-bond geometries of *X*_3_ synthons in C_6_Cls_6_ (HCB), C_6_Cl_5_OH (PCP), C_6_Br_6_ (HBB) and C_6_Br_5_OH (PBP) α is the angle between the CD⋯CC and internuclear directions. Parameters with units: *d* (Å), angles (°), *RR* (dimensionless). vdW radii = 1.75 and 1.85 Å for Cl and Br atoms, respectively.

Molecule	*X*(δ^+^)⋯(δ^−^)*X*†	*d*	*RR*	ζ_1_	ζ_2_	|Δζ|	α
C_6_Cl_6_	Cl_1_^i^⋯Cl_2_^ii^	3.4466 (1)	0.98	175.0	116.7	58.3	7.6
	Cl_2_^ii^⋯Cl_3_^iii^	3.4701 (1)	0.99	174.7	124.2	50.5	9.3
	Cl_3_^iii^⋯Cl_1_^i^	3.6662 (1)	1.05	171.2	123.3	47.9	7.3
C_6_Cl_5_OH	Cl_4_^i^⋯Cl_2_^ii^	3.4095 (1)	0.97	175.1	78.3	96.8	13.5
	Cl_2_^ii^⋯Cl_3_^iii^	3.6476 (2)	1.04	126.4	110.3	16.1	10.8
	Cl_3_^iii^⋯Cl_4_^i^	3.6197 (2)	1.03	177.5	122.1	55.4	5.5
C_6_Br_6_	Br_1_^i^⋯Br_2_^ii^	3.5412 (2)	0.96	174.2	115.1	59.1	12.2
	Br_2_^ii^⋯Br_3_^iii^	3.5551 (4)	0.96	176.7	123.8	52.9	10.5
	Br_3_^iii^⋯Br_1_^i^	3.7761 (2)	1.02	171.2	122.8	48.4	10.8
C_6_Br_5_OH	Br_2_^i^⋯Br_3_^ii^	3.5066 (2)	0.95	173.3	117.3	56.0	10.3
	Br_3_^ii^⋯Br_4_^iii^	3.6576 (2)	0.99	173.0	122.6	50.4	9.0
	Br_4_^iii^⋯Br_2_^i^	3.7127 (3)	1.00	175.7	120.7	55.0	11.7

† Symmetry codes. C_6_Cl_6_: (i) *x*, *y*, *z*; (ii) 1/2 − *x*, 1/2 + *y*, 1/2 − *z*; (iii) −1/2 − *x*, −1/2 + *y*, 1/2 − *z*; (iv) −1 + *x*, 1 + *y*, *z*. C_6_Cl_5_OH: (i) *x*, *y*, *z*; (ii) *x*, 3 − *y*, −1/2 + *z*; (iii) −*x*, *y*, 1/2 − *z*; (iv) −*x*, 3 − *y*, −*z*. C_6_Br_6_: (i) *x*, *y*, *z*; (ii) 1.5 − *x*, 1/2 + *y*, 1/2 − *z*; (iii) −1/2 + *x*, 1/2 − *y*, 1/2 + *z*; (iv) 2 − *x*, 1 − *y*, −*z*. C_6_Br_5_OH: (i) *x*, *y*, *z*; (ii) 2 − *x*, 1/2 + *y*, 1 − *z*; (iii) 1 − *x*, 1/2 + *y*, 1 − *z*; (iv) −1 + *x*, 1 + *y*, *z*.

**Table 4:** Structural parameters, topological and energetic properties at BCPs, and estimated interaction energies for *X*^δ+^⋯^δ−^*Y* (*X* = H, I; *Y* = O, Cl) interactions in the V-type motifs present in the crystal structures of IDT and PCP α is the angle between the CD⋯CC and internuclear directions. Parameters with units: *d* (Å), *RR* (dimensionless), ζ_1_ and ζ_2_ (°), α (°), and topological properties as in Table 2 ▸. For individual interactions, −*E*_int_ (kJ mol^−1^) is estimated as (left) −*V*/2 and (right) −0.556*V* − 2.697 [for XB interactions involving I atoms, see Bauzá & Frontera (2020 ▸)]. The interaction energies in IDT and PCP dimers have been calculated at the B3LYP-D3/Def2TZVPP level of theory: values (in kJ mol^−1^) correspond to −*E*_int_system_/−*E*_int_system_corrected_BSSE_/−Σ*V*/2/−Σ*E*_int_(Bauzá & Frontera, 2020 ▸)_. vdW radii = 1.20, 1.52, 1.75 and 1.98 Å for H, O, Cl and I atoms, respectively.

Compound	*X*(δ^+^)⋯(δ^−^)*Y*	*d*	*RR*	ζ_1_	ζ_2_	ρ	∇^2^ρ	*G*	*V*	|*V*|/*G*	−*E*_int_	α
IDT	C2—H2⋯O1—C1	2.235	0.82	158.7	137.9	0.089	1.25	28.4	−22.6	0.80	11.3/–	8.5
	C3—I1⋯O1—C1	2.9342 (3)	0.84	170.3	135.5	0.112	1.49	35.6	−30.6	0.86	15.3/14.3	1.8
	Dimer†*E*_int_										33.4/31.6/26.6	
PCP	O1—H1⋯O1—C6	2.018	0.74	151.3	125.4	0.136	1.69	43.6	−41.2	0.95	20.6/–	6.5/15.7
	O1—H1⋯Cl5—C5	2.943	1.00	114.7	89.3	0.038	0.51	10.8	−7.7	0.71	3.8	7.0
	Dimer *E*_int_										24.8/22.3/24.4	

† For the calculation of the interaction energy of the motif, the IDT trimer is considered to be a dimer formed by the molecule involving an O atom in the V-type motif and the supra-molecular rigid unit formed by the two assembled molecules that involve the I and H atoms in the V-type motif.

**Table 5:** Structural parameters, topological and energetic properties at BCPs, and estimated interaction energies for intramolecular interactions *X*(δ^+^)⋯(δ^−^)*Y* (*X* = H, C, Se, Br; *Y* = O, C, Br) in PBP, GOHJAQ and AHEQIN α is the angle between the CD⋯CC and internuclear directions. Parameters with units: *d* (Å), ζ_1_ (°), ζ_2_ (°), α (°), topological properties as in Table 2 ▸. Interaction energies −*E*_int_ (kJ mol^−1^) are estimated as in Tables 2 ▸ and 4 ▸.

Compound	*X*(δ^+^)⋯(*δ^−^*)*Y*	*d*	*RR*	ζ_1_	ζ_2_	ρ	∇^2^ρ	*G*	*V*	|*V*|/*G*	−*E*_int_	α
PBP	O—H⋯Br—C	2.156	0.71	146.1	62.9	0.235	1.70	58.2	−70.1	1.20	35.1/–	15.0
AHEQIN	C—Se⋯O—C	2.561	0.75	170.3/76.6	103.0	0.212	2.35	64.9	−65.7	1.01	32.9/27.0	3.1
	C—H⋯C(π)—N	2.354	0.81	110.7	90.3	0.101	1.22	28.9	−24.6	0.85	12.3/–	13.8
GOHJAQ	C—Br⋯O—C	2.909	0.86	174.5	115.7	0.101	1.38	31.9	−26.4	0.83	13.2/12.0	5.1
	C—C(π)⋯Br—C	3.516	0.99	98.4	134.1	0.040	0.46	10.1	−7.6	0.76	3.8/-	12.9
