# Supplementary material for: The origin of synthons and supramolecular motifs: beyond atoms and functional groups
Source: IUCrJ. 2025 Apr 7;12(Pt 3):334–57. doi: 10.1107/S2052252525001447 (PMC12044848; doi:10.1107/S2052252525001447)
Supplement: Supplementary file 3 [file m-12-00334-sup3.pdf]

IUCrJ

Volume 12 (2025)

Supporting information for article:

The Origin of Synthons and Supramolecular Motifs: Beyond Atoms and Functional Groups

Rahul Shukla, Emmanuel Aubert, Mariya Brezgunova, Slimane Dahaoui, Sébastien Lebègue, János G. Ángyán, Julien Lieffrig, Marc Fourmigué and Enrique Espinosa

Table S1 Crystallographic data and experimental details for **IDT**. Spherical and theoretical multipolar refinement were performed separately.

Crystal structure	
empirical formula	C_3HIOS_2
formula weight (g/mol)	244.06
temperature (K)	100(2)
source / wavelength (Å)	MoK_α / 0.71073
crystal system / space group	Triclinic / *P*1
unit cell dimensions *a* (Å), *b* (Å), *c* (Å)	4.0936(1), 5.6360(2), 6.6467(2)
α (°), β (°), γ (°)	103.946(2), 91.017(2), 91.983(2)
volume (Å^3)	148.687(7)
Z	1
calculated density (g/cm^3)	2.728
absorption coefficient μ (mm^{-1})	5.96
crystal size (mm^3)	0.11 × 0.06 × 0.03
F(000)	112
Data collection	
$(\sin \theta/\lambda)_{max}$ (Å^{-1}): exp/theor	1.28 / 1.0
index range	$-10 \leq h \leq 10$
	$-14 \leq k \leq 14$
	$-16 \leq l \leq 17$
reflections collected	66473
independent reflections	10013
R_{int}	0.082
completeness to $(\sin \theta/\lambda)_{max}$ (%)	97.6
absorption correction	analytical
min/max transmission	0.65 / 0.88
Spherical refinement data	

refinement method	F^2
data/restrains/parameters	10013/ 3/ 65
largest diff peak/hole (eÅ^{-3})	2.42 / -3.20
GOF on F^2	1.137
R (all data)	0.0484 (10013)
R ($I > 2\sigma(I)$)	0.0353(8709)
w$R(F)$	0.0680
Theoretical Multipolar refinement data	
refinement method	F
data: $I > 3\sigma(I)$/total [a]	2489
N_{ref} / N_{var}	15.8
GOF on F [b]	0.023
$R(F)$	0.042
w$R(F)$	0.023

[a] The values correspond to theoretical (VASP calculations) data. [b]The low magnitude of the goodness-of-fit for theoretical calculations is due to the use of theoretical structure factors, for which the associated standard deviations are set to 1. In that case, the goodness-of-fit should converge towards 0 rather than 1.

Table S2 Structural and topological parameters for the Ch···X (X = Chal, I, O) chalcogen bonding interactions in the crystal structures of **IDT** and **SePA**.[a]

Ch(δ^+)···(δ^-)X [b]	d	RR	ρ	$\nabla^2\rho$	λ_1	λ_2	λ_3	G	V	-E_{int}	$\|V\|/G$
$S_1^{i}\cdots S_2^{iii}$	3.8308(1)	1.06	0.034	0.33	–0.05	–0.04	0.42	7.1	–5.2	2.6	0.74
$Se_1^{i}\cdots Se_1^{ii}$	3.8220(2)	1.01	0.051	0.37	–0.10	–0.09	0.56	9.0	–7.8	3.9/5.3	0.83
			0.047	0.37	–0.09	–0.09	0.54	8.6	–7.2	3.6/5.1	0.84
$S_2^{iii}\cdots I_1^{i}$	3.8035(3)	1.01	0.059	0.51	–0.14	–0.12	0.77	12.1	–10.3	5.1	0.85
$Se_1^{i}\cdots O_2^{ii}$	3.3552(1)	0.98	0.049	0.62	–0.11	–0.11	0.84	13.3	–9.8	4.9/6.0	0.74
			0.049	0.62	–0.11	–0.11	0.85	13.3	–9.7	4.9/6.0	0.73

[a] Structural parameters: interaction distance d(Å), RR (reduction ratio) is defined as the Ch···Y distance over the sum of van der Waals radii (vdW radii = 1.52, 1.80, 1.90 Å and 1.98 Å for O, S, Se and I). Symmetry codes: **IDT**: (i) x, y, z; (iii) x, y, -1+z; **SePA**: (i) x, y, z; (ii) 1/2-x, -1/2+y, 3/2-z. The topological parameters ρ (eÅ^{-3}), $\nabla^2\rho$ (eÅ^{-5}), λ_i (eÅ^{-5}), G and V (kJ mol^{-1} bohr^{-3}) are calculated at BCPs of $\rho(\mathbf{r})$. -E_{int} (kJ mol^{-1}) values are

estimated as (left) -*V/2*, the constant being in bohr^3 units, and (right) -0.375*V + 2.366 (for ChB interactions involving Se-atoms, see Bauzá & Frontera, 2020). [b]First and second lines correspond to experimental and theoretical (VASP periodic calculations) data in case of **SePA**. For **IDT**, only theoretical multipolar refinement values are reported.

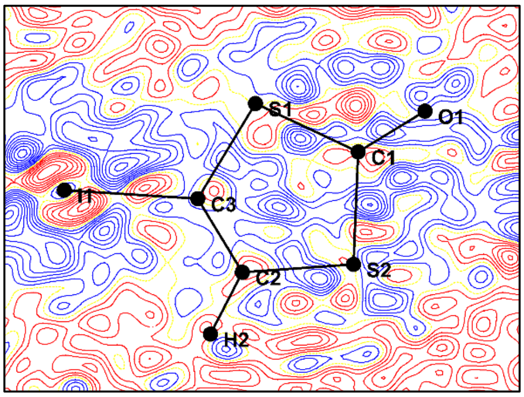

Figure S1 Experimental residual electron density map (resolution range $0 \leq (\sin\theta/\lambda) \leq 0.9$ Å^{-1}) after multipolar refinement for **IDT**. Contours are at 0.05 e Å^{-3} level (positive and negative values are in blue and red colours, respectively). The presence of several heavy atoms in a small organic molecule that crystalizes in the triclinic system with the non-centrosymmetric space group $P1$, lead to an experimental data set of limited quality. Despite several attempts were made to perform the experimental multipolar refinement, large magnitudes of residuals were present.
